# Using potential master regulator sites and paralogous expansion to construct tissue-specific transcriptional networks

**DOI:** 10.1186/1752-0509-6-S2-S15

**Published:** 2012-12-12

**Authors:** Martin Haubrock, Jie Li, Edgar Wingender

**Affiliations:** 1 Department of Bioinformatics, University Medical Center Göttingen, Goldschmidtstrasse 1, D-37077 Göttingen, Germany

## Abstract

**Background:**

Transcriptional networks of higher eukaryotes are difficult to obtain. Available experimental data from conventional approaches are sporadic, while those generated with modern high-throughput technologies are biased. Computational predictions are generally perceived as being flooded with high rates of false positives. New concepts about the structure of regulatory regions and the function of master regulator sites may provide a way out of this dilemma.

**Methods:**

We combined promoter scanning with positional weight matrices with a 4-genome conservativity analysis to predict high-affinity, highly conserved transcription factor (TF) binding sites and to infer TF-target gene relations. They were expanded to paralogous TFs and filtered for tissue-specific expression patterns to obtain a reference transcriptional network (RTN) as well as tissue-specific transcriptional networks (TTNs).

**Results:**

When validated with experimental data sets, the predictions done showed the expected trends of true positive and true negative predictions, resulting in satisfying sensitivity and specificity characteristics. This also proved that confining the network reconstruction to the 1% top-ranking TF-target predictions gives rise to networks with expected degree distributions. Their expansion to paralogous TFs enriches them by tissue-specific regulators, providing a reasonable basis to reconstruct tissue-specific transcriptional networks.

**Conclusions:**

The concept of master regulator or seed sites provides a reasonable starting point to select predicted TF-target relations, which, together with a paralogous expansion, allow for reconstruction of tissue-specific transcriptional networks.

## Background

Regulation of transcription is mediated through complex arrays of transcription factor binding sites (TFBSs), which constitute promoter and enhancer regions. In spite of the advent of high-throughput approaches to identify TFBSs in a given cellular context, the available information, most comprehensively collected in the TRANSFAC^® ^database [1], is still fragmented and biased with regard to the systems selected. Consequently, any transcriptional network reconstructed from the available experimental data is highly incomplete. This situation deteriorates further when filtering such a transcriptional "reference" network for gene expression data in order to generate tissue-specific networks. Therefore, constructing comprehensive gene regulatory networks still depends on reliable algorithms for predicting individual TFBSs as a basis for inferring TF-target gene relations. These predictions, however, depend on the availability of information about the DNA-binding specificity of ideally all TFs encoded by a genome. Unfortunately, we are far from this ideal situation, so that we can do such predictions only for a subset of, e.g., human TFs. Although promising methods have been reported for inferring DNA-binding specificities by homology modeling [2,3], the required 3D structures of TF-DNA complexes are known for only a minority of factors.

Recent studies have applied high-throughput approaches to map active promoters and enhancers in a particular cellular context by capturing epigenetic characteristics such as specific histone methylation patterns [4]. However, it still has to be revealed what the exact regulation of a given gene is, i.e. which functional TFBSs are there in its regulatory regions, and which is the original signal that flags a promoter region as such. Conceivably, the recently published concepts about master transcription factors [5] or pioneer transcription factors [6] may provide a clue to this problem.

In this study, we started from the following related working model as hypothesis: In the genome of a given higher eukaryotic cell, promoter sequences have to be "flagged" in order to be recognizable by the transcription machinery. Each of these flags is realized by a high-affinity TFBS, which, due to its functional importance, is generally conserved among genomes that are phylogenetically not too distant. These high-affinity and conserved sites serve as nucleation centers, or "seeds", to govern the proper assembly of TFs at one promoter, which also involves a set of additional transcription factors with binding sites of decreasing affinity and acting in a concomitantly optional manner.

## Methods

### TFBS prediction

We started from 35,750 RefSeq-annotated human promoter regions (UCSC track refGene, Apr. 14, 2010, hg19) which are linked to 21,532 unique genes. We selected the 1-kb upstream regions based on the RefSeq annotation to cover the corresponding human promoter regions. We retrieved ortholog promoter regions from mouse, dog, and cow genomes from the 46_WAY_MULTIZ_hg19 whole genome alignments provided by UCSC for 46 vertebrates using UCSC/Galaxy [7]. The corresponding sequence builds are hg19, mm9, canFan2, and bosTau4. Gaps resulting from the multiple genome alignment were removed from the promoter sequences. Potential transcription factor binding sites (TFBS) were then identified using all available vertebrate matrices (854 PWM) of the TRANSFAC matrix library (release 2009.4) and the program Match^™ ^[8]. We applied all vertebrate matrices using default minFN ("minimize false negatives") thresholds in order to retrieve almost all potential transcription factor binding sites that have at least the quality of the used TFBS which are given in the corresponding matrix [8]. The predictions were then mapped back to the whole genome alignments. We next filtered for conserved TFBS predictions: a conserved TFBS has to start or end at a non-gap symbol in the corresponding promoter alignment. Finally we ranked all conserved TFBSs according to their Match score and selected the top-ranking 1%, 2%, 3%, 5%, etc for evaluations. The 100% profile comprises all conserved TFBSs that were identified with minFN thresholds. For further analyses of the network characteristics, the top-ranking 1% predicted binding sites for each matrix were used.

### From predictions to gene regulatory network

Using the TRANSFAC library we ended up with a list of predicted transcription factor binding sites related to the TRANSFAC matrix identifiers. To build gene regulatory networks we translated these matrix identifiers, which are linked to lists of related species-specific proteins, to official human gene symbols.

For "paralogous expansion", we used our new Human Transcription Factor Classification to construct gene regulatory networks (http://www.bioinf.med.uni-goettingen.de/projects/tfclassification/). This collection classifies human transcription factors into families and subfamilies mainly based on the sequence similarities of their DNA-binding domains (DBDs). Since at the lowest classification level, the DBDs are usually extremely similar, the DNA-binding specificities can be assumed to be nearly identical as well. We therefore expanded all TF-target links to all members of the corresponding TF (sub-)family, for which no matrix is as yet available.

### Evaluation of conserved binding site prediction

The verification of the predicted binding sites was done using experimentally identified regulatory regions from the Encode project [9]. ENCODE provides a regulatory super-track as a downloadable file. This archive is summarizing all transcription factor ChIPseq experiments which have been done within the ENCODE project based on the human genome build 37 (hg19). Altogether whole genome binding sites and their genomic coordinates are available for more than 140 different human transcription factors. They were used to evaluate our TFBS and the inferred TF-target predictions by computing the True Positive (TP), False Positive (FP), False Negative (FN), and True Negative (TN) rates for some human transcription factors. If a predicted TFBS is found in a ChIP-seq region as well, we count it as a TP. If a TFBS prediction is not detected by a ChIPseq experiment this is an FP result. An FN result is obtained when a ChIPseq region is overlapping with a potential promoter region (including the fragment of overlapping the promoter regions at least with 500 nucleotides), but we don't predict a TFBS for this situation. A TN result is related to a situation, where we neither predicted a TFBS nor a ChIPseq region was found. Using these statistical measurements we determine the Positive Predictive Value (or precision; PPV = TP/(TP+FP)), Specificity (Spec = TN/(TN+FP)), and the True-Positive-Rate (TPR = TP/(TP+FN); also: sensitivity or recall) to detect the accuracy of a ChIP-seq evaluation.

### Tissue-specificity of transcription factors

Based on UniGene [10] we have downloaded the gene expression profiles for 8 different tissues: brain, heart, kidney, liver, ovary, prostate, spleen, testis.

## Results

### Reconstruction of the transcriptional network through predicted TFBSs

Previous studies have shown that sequence conservation can improve transcription factor binding site predictions [11,12]. Therefore, we combined standard PWM scanning with a four species conservation filtering to identify potential TFBSs and, on this basis, to infer TF-target gene relations for a comprehensive reference transcriptional network (RTN). With this strategy (see Methods for details), we predicted 4,3*10e7 TFBS which are conserved among these four species (hg19, mm9, canFam2, bosTau4). These predictions are linked to 16,900 unique human gene symbols. 47.3% of all human promoters (35,750 RefSeq-annotated human promoter regions) share at least one conserved predicted binding site with the mouse, dog, and cow species. When selecting only the best 1% predictions of each TRANSFAC matrix we found that 15,619 genes (43.7%) share a conserved, high-scoring binding site. Altogether, we ended up with 490,277 TFBS predictions.

### Paralogous expansion of the transcription network

We used a fundamentally revised version of an earlier transcription factor classification, based on their DNA-binding domains [13], to identify groups of TFs that share DNA-binding specificity to the largest extent possible. They may be regarded as paralogs, resulting from early gene duplication events (Wingender, manuscript in preparation). This classification scheme comprises four abstraction levels: superclass, class, family, and (optionally) subfamily. Whenever one member of a bottommost clade (family or subfamily) has a TRANSFAC matrix associated, all potential binding sites and, thus, target genes predicted for this TF were copied to all other clade members. This expansion of the transcriptional reference network led to an increase of the TF genes from 442 to 742 (by 67.9%), and increased the number of directed edges in the network from 277,661 to 728,667 (by 162.4%) (Additional file 1, first line of the table). The expansion approach was also cross-validated for those cases where distinct (sub)family members had different TRANSFAC matrices associated (data not shown).

### Validation of the reconstructed network

**Figure 1:**
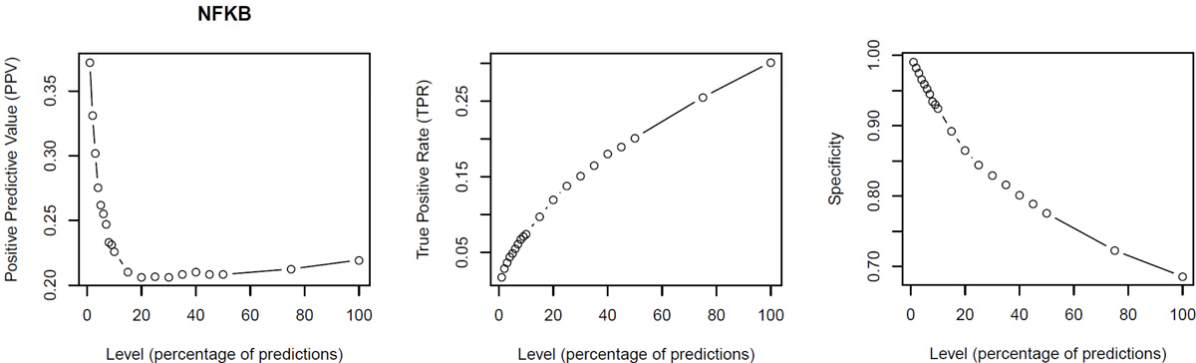
**Validation of NF-κB sites**. Validation of predicted NF-κB sites with ChIPseq data sets from ENCODE. Given are the positive predictive value (PPV = TP/(TP+FP)), the true positive rate (TPR = TP/(TP+FN)), and the specificity (SPC = TN/(TN+FP)) for all profiles ranging from the 1% top-ranking down to 100% of the predictions made (see Methods for details).

For 22 different transcription factors we investigated the performance of our predictions for 20 different quality levels by gradually increasing the percentile of highest scoring potential TFBSs accepted, ranking from the 1% best sites down to 100%, which are all conserved sites that could be predicted with the minFN threshold. For a number of TFs, for which experimental ChIPseq data were available at ENCODE, we determined the Positive Predicted Value (PPV), the True Positive Rate (TPR) and the Specificity for the whole range of quality levels (from the 1% best predictions to 100% of all conserved TFBSs; see Methods section for details). Figure 1 is demonstrating these three values for transcription factor NF-κB (NFKB; see Additional File 2 for all plots). For all 22 TFs studied so far, we observed a very high specificity for the 1% selection. For E2F1, E2F4, and E2F6, the specificity was about 80%, whereas it was clearly above 90% for all other 19 TFs (BRCA1, CTCF, ELF1, ETS1, FOXA1, GATA1, GATA2, GATA3, HEY1, IRF1, IRF3, NANOG, NFKB, PAX5, POU5F1, RXRA, SP1, TFAP2A, YY1). For less stringent predictions, i.e. when proceeding towards the 100% level, the specificity decreases continuously. In contrast, the TPR (sensitivity) is increasing from 1% to 100% selection. We observed heterogeneous profiles for the PPV, with usually the highest value for the 1% profile (up to ~82% in the case of CTCF or ~65% for YY1). In some cases, reducing the stringency of filtering led to a disproportionate increase of the TP and, thus, to an increase of the PPV. This indicates that a very high number of "real" sites are "suboptimal", i.e. match with the matrix/matrices used only at relatively low scores. In the respective contexts, these sites may have evolved to exhibit only moderate affinity instead of strongest conceivable binding. Some TFBS predictions show relatively low PPVs, which may be regarded as high numbers of false positives. However, this perception will be challenged by further investigations (see next paragraph).

Altogether, we decided to work furtheron with the 1% profiles and the resulting networks.

### Revisiting false positives

**Figure 2:**
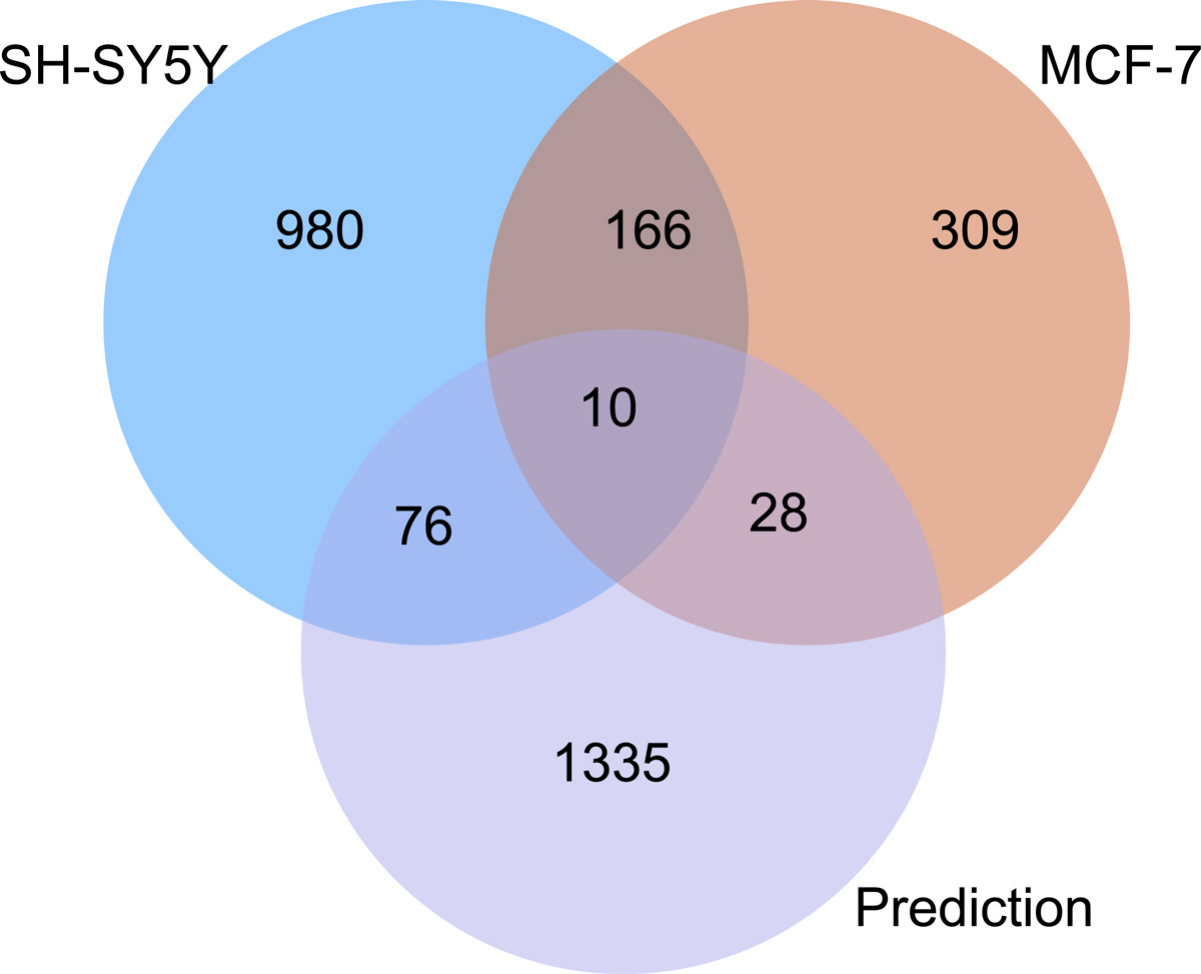
**Different ChIPseq data sets show little overlap**. Predicted high-affinity (1% top-ranking), highly conserved binding sites for GATA-3 (purple circle, bottom), and GATA-3 ChIPseq fragments from SH-SY5Y cells (blue circle, top-left) or from MCF7 cells (brown circle, top-right), overlapping with 1kb upstream sequences, were checked for the respective intersections.

The PPVs obtained (see above and Additional File 2) seem to indicate that there is still a considerable number of FP even under the most stringent conditions (1% highest scoring conserved TFBSs). To explore this a bit further, we determined again the TP, FP, TN and FN rates of our 1% top-ranked predictions for five TFs (GATA3, MYC, JUN, MAX, FOS), but using now two independent ENCODE ChIPseq data sets for each of these TFs. These ChIPseq data indicate for each of these factors the binding sites that are used in different cell lines, representing different tissues in all these cases. Figure 2 shows for GATA-3 (see Additional file 3 for the remaining TFs), that two independent ChIP data sets yield quite different TP and FP numbers, with relatively little overlap: they have only 176 targets in common and predicted by our approach, and even the overlap between the two experimental data sets comprises only a minority of the proven sites. We obtained the same picture for four further TFs for which we could retrieve new, independent data sets. Altogether, these results clearly show that whatever experimental data set is used for validating the predictions, a considerable number of alleged "false positive" predictions turns into TPs when the experimental basis broadens. In other words, determining the FP rate with only a limited set of experimental data highly overrates this error type.

In the Venn diagram of Figure 2, the overlap between the predictions and any experimental data set may nevertheless appear small when compared with the overlap between the two ChIPseq data sets. It should be noticed, however, that we explicitly accepted a high number of False Negatives, as an unavoidable trade-off of the approach chosen here aiming at high-affinity and highly conserved sites only, regarded as potential master regulator or seed sites.

### Architecture of the reconstructed network

**Figure 3:**
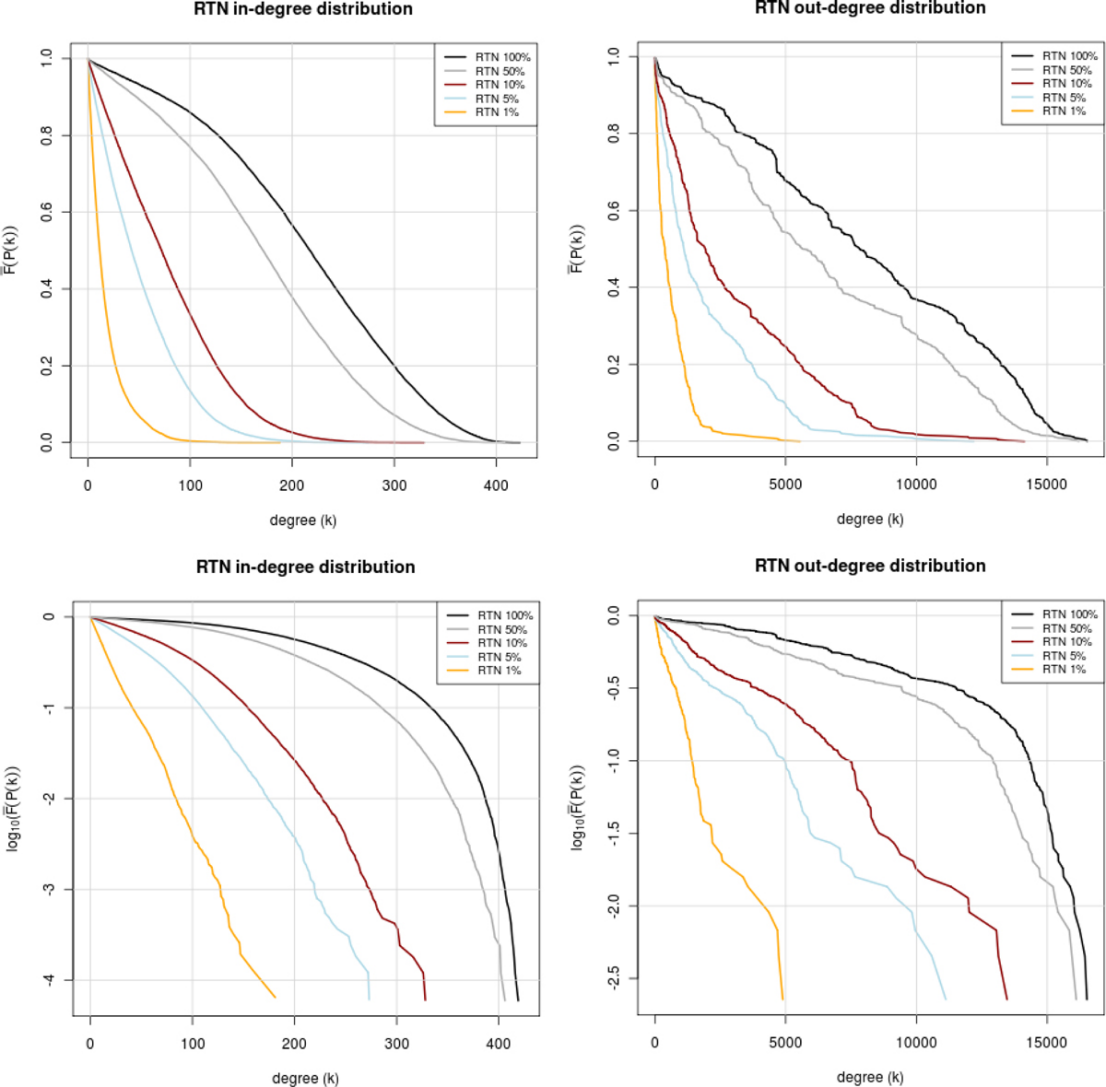
**Degree distributions in the reference transcriptional networks (RTNs) Inverse cumulative distribution of in- (left) and out-degree (right), in linear (top) and semi-logarithmic plot (bottom)**. The distributions are shown for the reference transcriptional networks (RTN) reconstructed with the indicated percentiles of top-ranking predicted and conserved TFBSs.

For an initial analysis of the reference transcriptional network (RTN) obtained by predicting high-affinity, highly conserved sites and subsequent paralogous expansion, we investigated the distribution of in- and out-degrees. Since especially the out-degrees can adopt very large values, but each degree class is extremely sparsely populated, we computed the inverse cumulative distribution function for the degree frequencies [14]. It was observed that both the RTN as well as the eRTN (expanded reference transcriptional network), when confining to the 1% highest scoring TFBS, show a clear exponential degree distribution. This is particularly obvious from the corresponding semi-logarithmic plots (Figure 3), where the correlation coefficients for a linear fitting of 1% profiles are -0.9985 and -0.9982 for the in-degrees and -0.9803 and -0.9846 for the out-degrees (RTN and eRTN, resp.).

**Figure 4:**
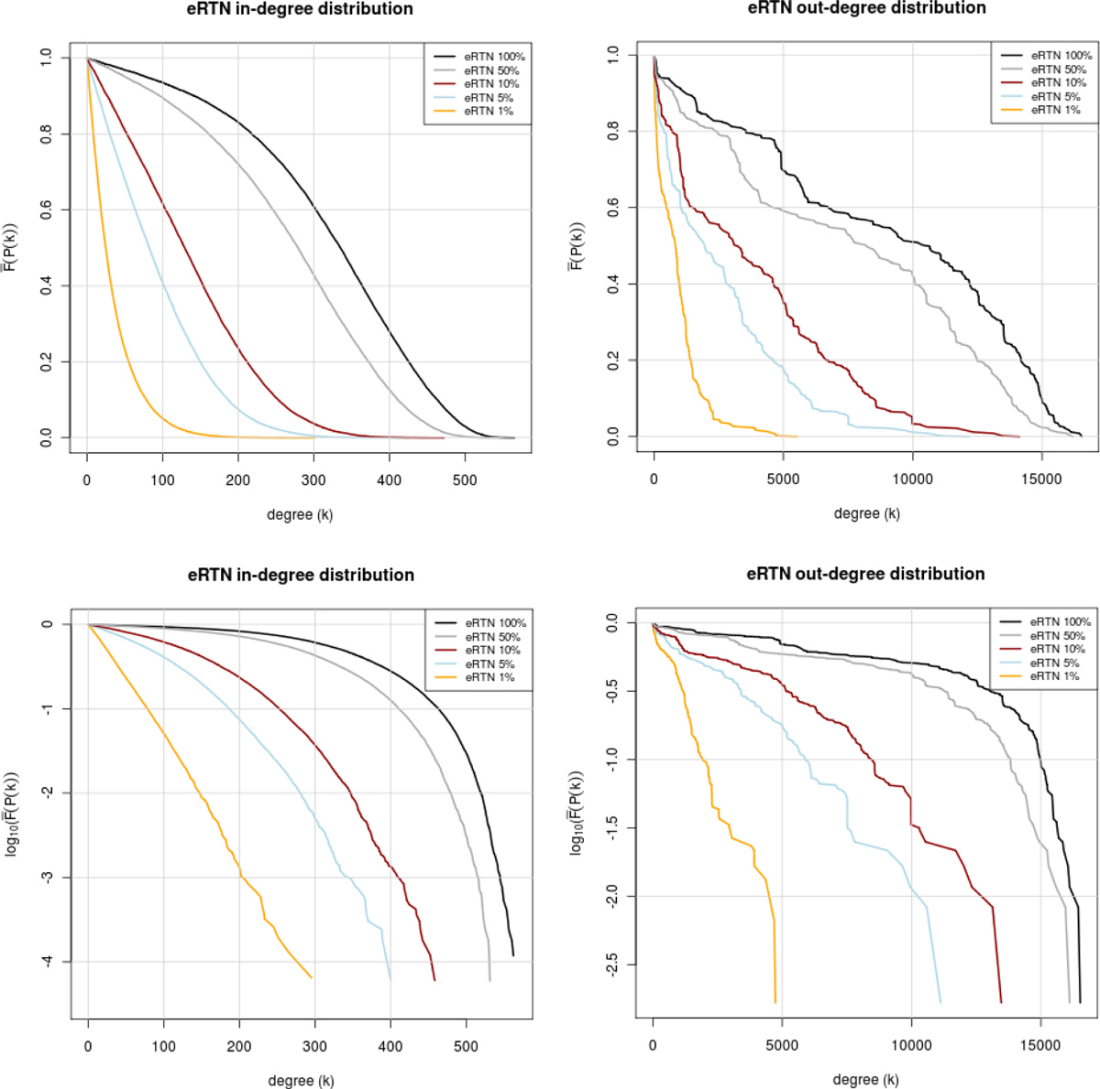
**Degree distributions in the expanded reference transcriptional networks (eRTNs)**. Inverse cumulative distribution of in- (left) and out-degree (right), in linear (top) and semi-logarithmic plot (bottom). The distributions are shown for the expanded reference transcriptional networks (eRTN) reconstructed with the indicated percentiles of top-ranking predicted and conserved TFBSs.

Relaxing the prediction constraints, i.e. proceeding from the 1% to the 100% profile, reveals the emergence of a shoulder around a degree of 100-200, possible indicating a superposition with a peaked distribution (in-degree) or very heterogeneous distribution until nearly the theoretical maximum (out-degree). This becomes even more visible when relaxing the constraint of conservativity (not shown). More important is that the expanded network (eRTN) in principle shows the same degree distributions, i. e. an exponential degree distribution in the 1% network (Figure 4). In the out-degree distribution, however, a number of peaks seem to be emerging in the less stringent networks.

### Reconstruction of tissue-specific transcription networks

**Figure 5:**
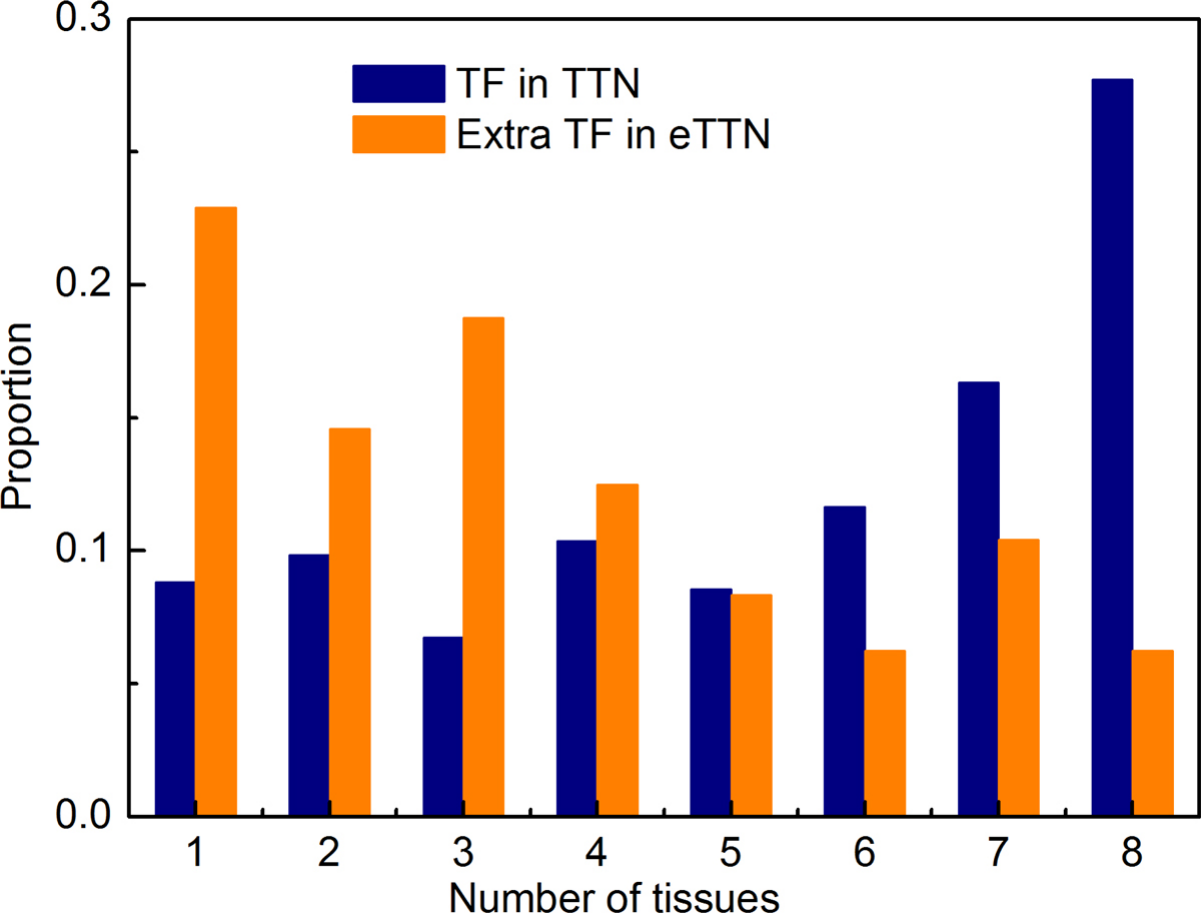
**Tissue distribution of the TFs in the tissue-specific transcriptional networks (TTNs) and the corresponding expanded networks (eTTNs)**. Shown are the portions of TF genes that appear in the indicated numbers of tissue-specific networks. The majority of TFs in the tissue-specific transcriptional networks (TTFs) that were derived from the RTN are expressed in many, if not all tissues studied (blue bars). In contrast, the newly added TF paralogs in the expanded TTNs (eTTNs) show a much more tissue-specific behaviour in being expressed in only one or few tissues (yellow bars).

Previously, we constructed eight tissue-specific transcription networks (TTNs for brain, heart, kidney, liver, ovary, prostate, spleen and testis) by reducing the RTN to those genes that are known to be expressed in the respective tissue [15]. Thus, regulatory edges survive this filtering only if both the regulator and the target gene are found to be expressed in the respective tissue. Here, we reconstructed the transcription networks for the eight tissues based on eRTN where the number of "active" TFs (i.e. those which have an out-degree >0) has nearly doubled (1.7-fold), and the number of directed edges has nearly tripled (2.6-fold; see above). Compared to the TFs in RTN, which are generally of low tissue-specificity (see Methods), the extra TFs in eRTN are mostly of high tissue-specificity (Figure 5). This indicates that the tissue networks extracted from eRTN are more comprehensive.

### Analysis of tissue-specific transcription networks

In general, the number of TFs in the expanded tissue transcription networks (eTTN) increased on average 1.5 times compared to those in the TTNs, whereas the number of nonTFs is almost constant (Additional file 1). This increase of TF numbers results in an even larger increase in the number of regulations (directed edges), which is on average 2.5 times higher than before the expansion, suggesting that the eTTNs are much more densely connected than the TTNs. It is noted that the increasing ratios of genes and regulations are generally consistent with the reference network and across the different tissues (Additional file 1). This indicates that the extra TFs in the eRTN, which are highly tissue specific (Figure 5), are a characteristic of all tissues studied so far.

The individual eTTN differ considerably in their sizes. By far the largest is the brain network, comprising 75% of the TF genes, 78% of the nonTF genes and 61% of the edges of the eRTN. At the other end of the scale, the spleen network shares with the eRTN only 31% of the TF genes, 38% of the nonTF genes, and 11% of the edges. On average, 41% of the regulations represented in the eRTN survive the tissue-specific filtering.

**Figure 6:**
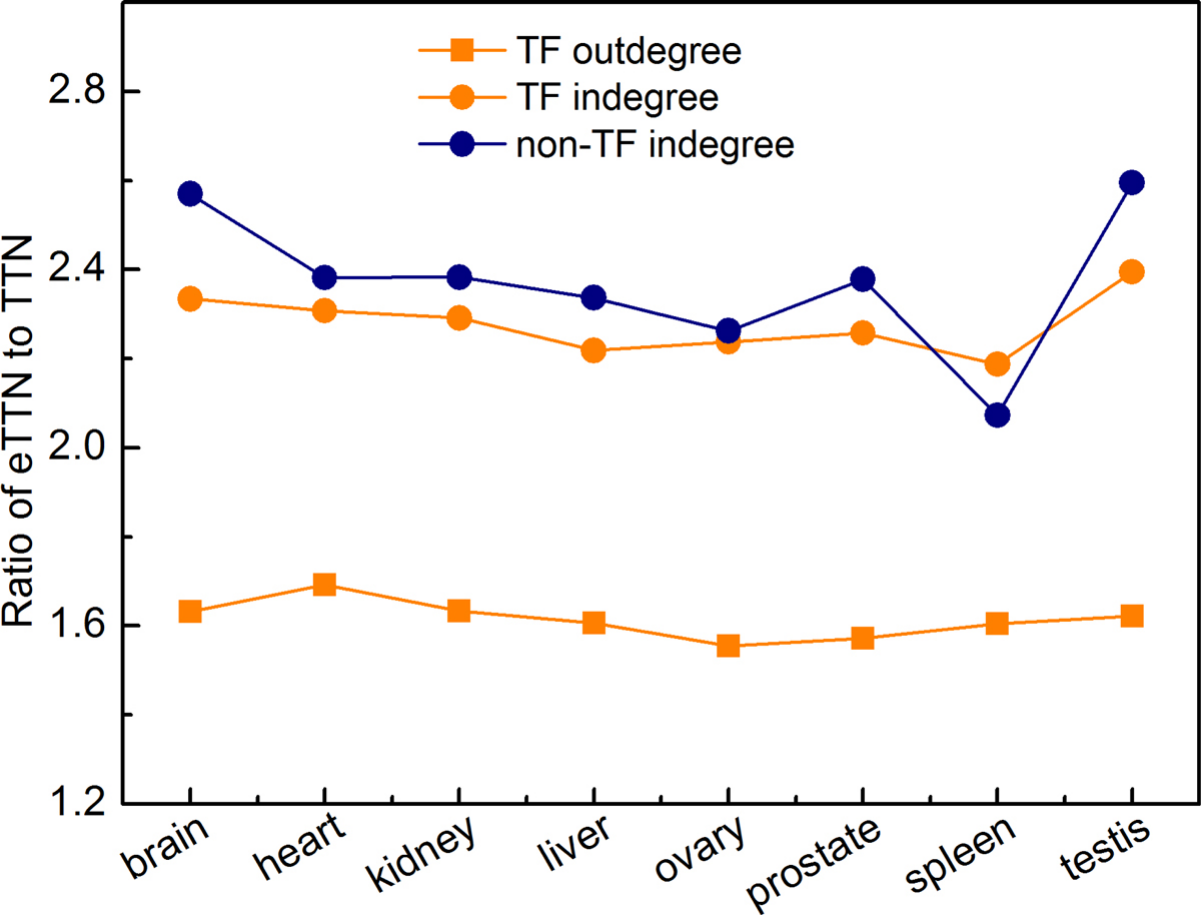
**Change of distribution in the tissue-specific networks by paralogous expansion**. The ratio of the respective degrees in the expanded tissue-specific networks (eTTNs) to those in the TTNs is shown for the individual tissues. The out-degree of TF genes changed moderately by the expansion in a nearly identical manner across all tissues (about 1.6-fold; yellow squares). In contrast, the in-degree increased considerably (about 2.3-fold), again similarly in all eTTNs (yellow dots). In general, the in-degree of non-TF genes increased slightly more, but with outliers for spleen (below) and testis (above the average; blue dots).

As to be expected, the average in-degrees of TFs and nonTFs increase much more than the average out-degrees of TFs in the eTTNs compared with the TTNs. On average, the in-degree rises around 2.3 times, but the out-degree increases only about 1.6 times (Figure 6; see also Additional file 4 for detailed numbers). This is understandable since many TFs are added in the eTTNs, which consequently results in a larger number of regulations that each target gene receives. The moderate increase of the out-degree is due to the fact that most of the newly added TFs had an out-degree well above the average.

Interestingly, the in-degree of TF genes is consistently about 50% larger than that of nonTF genes. This is true for the (e)RTN as well as for all (e)TTNs. This difference is only slightly diminished by the paralogous expansion (see Additional file 4).

However, such global increase of in- and out-degrees does not change the features of degree distributions of the eTTNs, which all show an exponential distribution of both in- and out-degree (Additional file 5).

### Case study on the heart-specific transcription network

It has been reported that during heart development, T-box transcription factors play a particularly important role [16]. Mutations in human *TBX *genes may result in cardiovascular malformations. Their gene products, the TBX factors, form a complex spatio-temporal pattern defining the identity of the different heart structures [17].

Human TBX factors are spread over five families, one of them comprising TBX2, TBX3, TBX4 and TBX5 (family 6.5.4 in our TF classification). Out of them, only TBX5 is associated with a positional weight matrix in TRANSFAC. However, it has been reported that for instance TBX3 can assist pluripotent reprogramming of embryonal fibroblasts, and is required to specify the atrioventricular system (AV) [18]. It prevents genes that are markers for other parts of the organ (e.g., for the chamber myocardium) to be expressed in AV, one of them is the gene of the atrial natriuretic factor (*NPPA*) [19]. It is noteworthy that after paralogous expansion, our heart network reveals *NPPA *as one of the more than 2000 target genes of TBX3, a relation that would have been lost otherwise.

## Discussion

With the efforts described in this paper, we made an attempt to reconstruct a realistic transcriptional network that (1) is void of false positive TF-target relations to the utmost extent possible, (2) includes as many regulator nodes (TFs) as possible, and (3) therefore provides a reasonable basis to reconstruct tissue-specific transcriptional networks. In order to minimize the number of false positive predictions, which is a well-known problem in computationally identifying TFBSs, we focused on highly conserved and high-affinity (by virtue of Match score) binding sites only to identify TF-target relations represented by the arcs in our reference network. Since we obtained relatively high PPV for most TFs, we are confident that the network we obtained is reliable. This is supported further by the observation that the FP rates we determined by comparing our predictions with experimental data sets, which always represent one (or very few) specific cellular situation(s), are highly overrated. Comparing experimental data sets for one and the same TF, but obtained from different cell types generally revealed minimal overlaps, confirming that many alleged FPs in fact may turn into true positives in a different cellular context, so that FP numbers are usually overrated. Rather, we suppose that most, if not all, high-affinity and conserved predicted TFBSs provide a regulatory potential that might be used in a certain cellular situation.

We are aware that our very stringent approach results in large numbers of false negatives, since many experimentally validated TFBS have a very low Match score and gain their functionality by the proper context of other elements. To include this kind of context, or the proper "syntax" of promoters, will be subject of further studies and an according updating of our network. Also the inclusion of enhancers will be a task for future work. We have observed that inclusion of conservativity as criterion does not well apply to enhancer regions, so that new concepts have to be developed for their identification and characterization.

Altogether, we are confident that the networks we have reconstructed reflect a relevant part of reality. This is also supported by the observed kind of degree distribution of the most stringent network, which follows a clear exponential law, as was to be expected at least for the in-degree distribution (see [20] and references cited therein) and from our own earlier observations for the out-degree distribution as well [21]. Relaxing the filter criteria leads to degree distributions with more random characteristics.

We have also shown that on the basis of such restrictive filtering, the networks can be reliably expanded by including related TFs and allow them to inherit all target relations, and with that the full out-degree, of already characterized (sub)family members. Since these newly added regulators predominantly provide tissue-specific regulatory information, this expanded network is a good basis to construct reliable transcriptional networks for individual tissues. A first overview revealed for these networks that their degree distributions follow the same rules as the reference network. In addition, first investigations have shown that also the hub composition of all these networks was comparable. Finally, we could show that in the particular case of heart development, paralogous expansion was able to rescue target genes for a specific transcription factor (TBX3), which otherwise would not have been amenable in the corresponding tissue-specific network.

## Conclusions

A paralog-expanded transcriptional network has been constructed based on the knowledge of master regulator or seed sites. It has been shown that the paralogous expansion provides as reliable basis to reconstruct tissue-specific transcription networks. The obtained networks show the expected statistical and topological characteristics. A first case study additionally provided biological evidence for the reliability and usefulness of these networks in including regulatory information which would have been missed without this expansion. From that we conclude that our approach to construct transcriptional network is valid and provides a solid ground for further studies, in particular with regard to the analysis of regulatory processes, e.g. the mechanisms governing cell differentiation.

## List of abbreviations

ChIP: chromatin immunoprecipitation; eRTN: expanded reference transcriptional network; eTTN: expanded tissue-specific transcriptional network; FN: false negative; FP: false positive; PPV: positive predictive value; PWM: positional weight matrix; RTN: reference transcriptional network; TF: transcription factor; TFBS: transcription factor binding site; TN: true negative; TP: true positive; TTN: tissue-specific transcriptional network.

## Competing interests

The authors declare that they have no competing interests.

## Authors' contributions

MH carried out the computational analysis of transcription factor binding sites, constructed the reference networks, did the network analyses and drafted parts of the manuscript. JL carried out the construction of the tissue-specific transcriptional networks and their analyses and drafted parts of the manuscript. EW conceived of the study, participated in its design and coordination and drafted the manuscript. All authors read and approved the final manuscript.

## Supplementary Material

Additional file 1 **Network statistics**. Given are the numbers of vertices (split by TF and nonTF genes) and edges in the transcriptional networks without or with expansion, for the reference network as well for the tissue-specific networks. Click here for file

Additional file 2 **Prediction validations**. In this file, plots of positive predictive value (PPV), true positive rate (TPR) and Specificity are given for all TFs where sufficient experimental data have been made available by the ENCODE project. Validation has been made in each case for all prediction profiles from the 1% top-ranking sites down to 100% of the predictions for conserved binding sites. Click here for file

Additional file 3 **Revisited false positives**. Venn diagrams of comparing independent experimental datasets for TFBSs within the -1kb regions with each other and the predictions done in this study. Click here for file

Additional file 4 **Degree statistics**. The table indicates the average out-degree and in-degree of TF genes as well as the average in-degree of nonTF genes (NTF) for both the reconstructed transcriptional networks (reference network and tissue-specific networks) as well as the transcriptional network expanded by related TFs. It also shows the ratios of the corrresponding values for the expanded and the non-expanded networks (eTN/TN). Click here for file

Additional file 5 **Degree distributions of tissue-specific transcriptional networks**. Inverse cumulative in- and out-degree distributions of the tissue-specific transcriptional networks (TTNs) before and after paralogous expansions. Click here for file
