# Supplementary material for: Using potential master regulator sites and paralogous expansion to construct tissue-specific transcriptional networks
Source: BMC Syst Biol. 2012 Dec 12;6(Suppl 2):S15. doi: 10.1186/1752-0509-6-S2-S15 (PMC3521180; doi:10.1186/1752-0509-6-S2-S15)
Supplement: Additional file 1 — Network statistics. Given are the numbers of vertices (split by TF and nonTF genes) and edges in the transcriptional networks without or with expansion, for the reference network as well for the tissue-specific networks. [file 1752-0509-6-S2-S15-S1.pdf]

Additional file 1

Network statistics

Network type	Transcriptional networks			Expanded transcriptional networks		
	Vertices (TF)	Vertices (nonTF)	Edges	Vertices (TF)	Vertices (nonTF)	Edges
Reference	442	15177	277661	742	15107	728667
Brain	343	11750	167560	555	11831	442658
Heart	230	7496	69185	334	7602	169985
Kidney	295	9865	118145	447	10044	292301
Liver	253	8662	88575	380	8787	213675
Ovary	240	7783	78274	366	7986	185617
Prostate	265	9099	99767	411	9136	243260
Spleen	161	5266	33962	230	5728	77877
Testis	282	10468	119221	468	10629	320805

Given are the numbers of vertices (split by TF and nonTF genes) and edges in the transcriptional networks without or with expansion, for the reference network as well as for the tissue-specific networks.
