# Supplementary figures and images for: Using potential master regulator sites and paralogous expansion to construct tissue-specific transcriptional networks
Source: BMC Syst Biol. 2012 Dec 12;6(Suppl 2):S15. doi: 10.1186/1752-0509-6-S2-S15 (PMC3521180; doi:10.1186/1752-0509-6-S2-S15)

## BRCA1

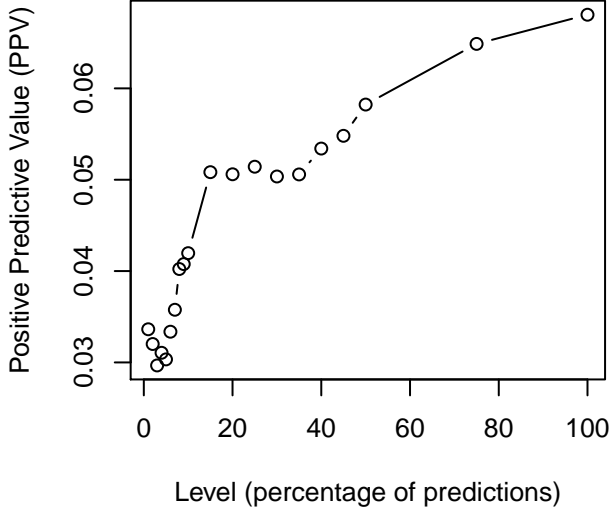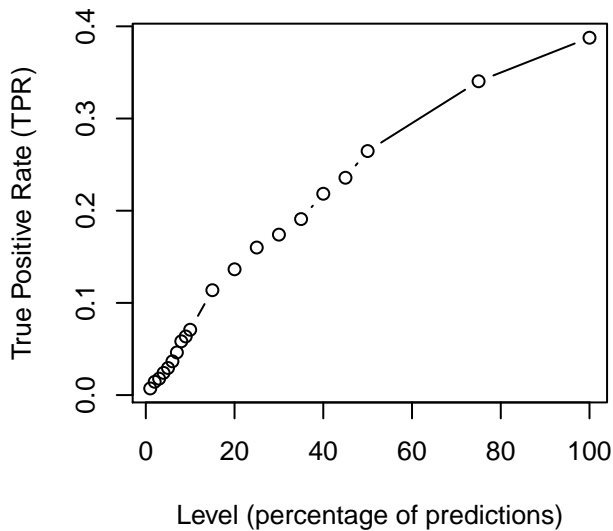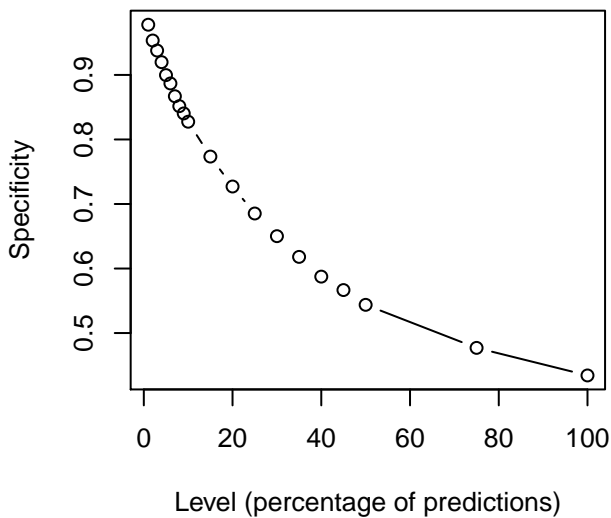

## CTCF

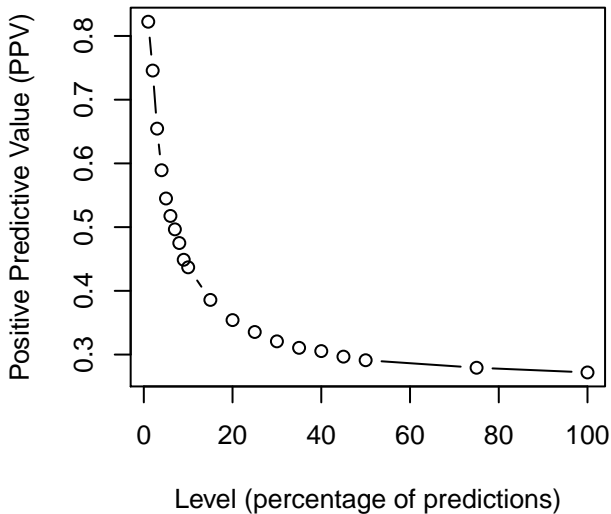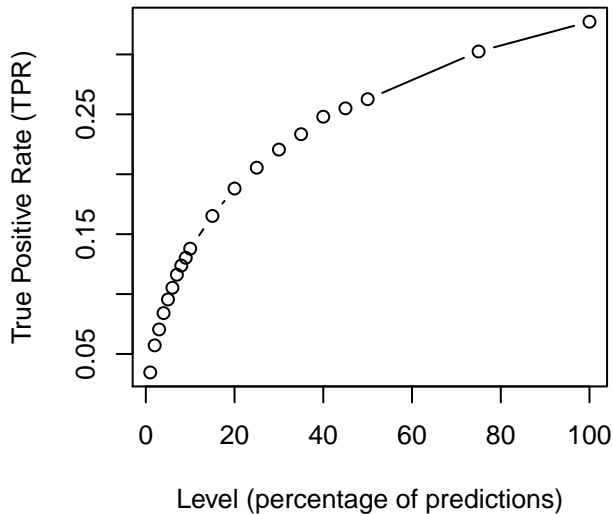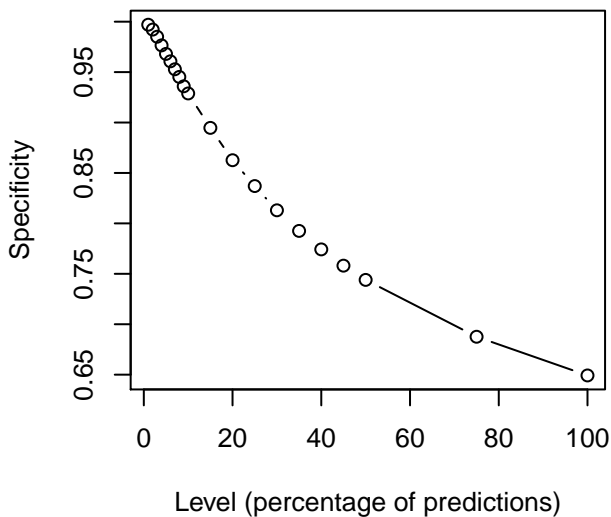

## E2F1

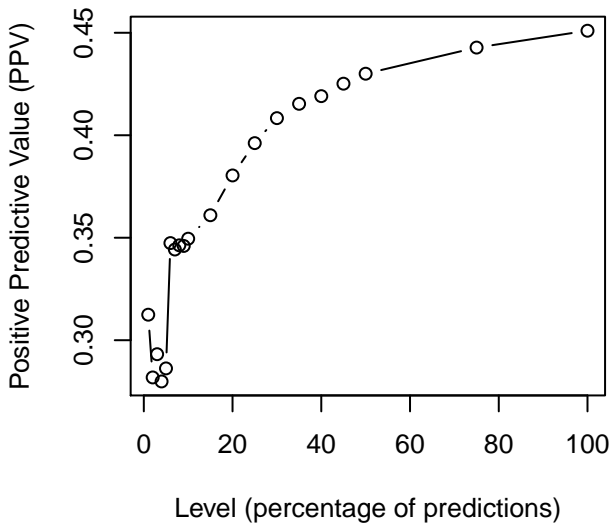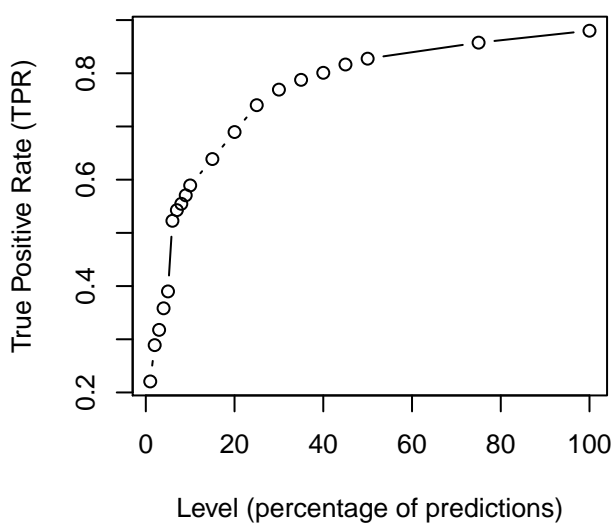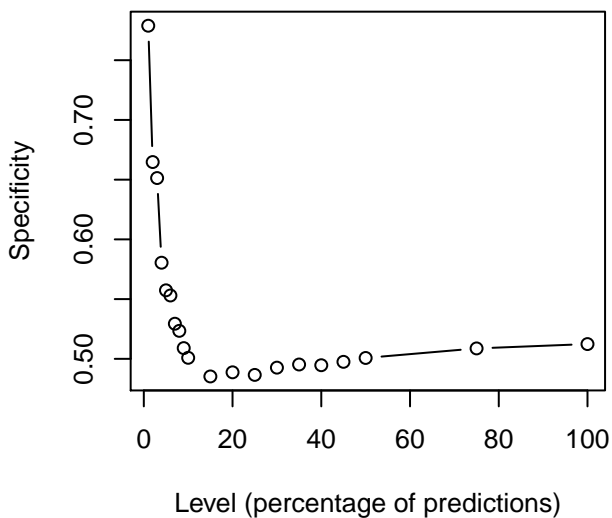

**E2F4**

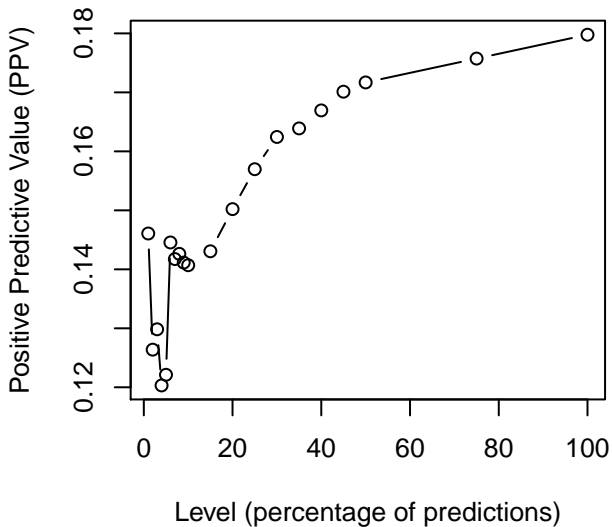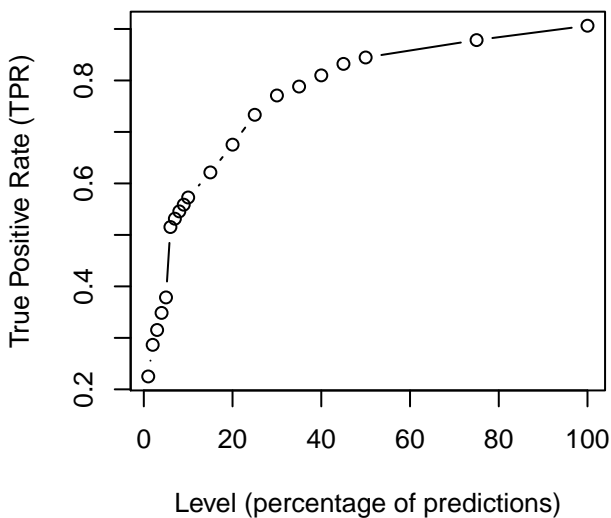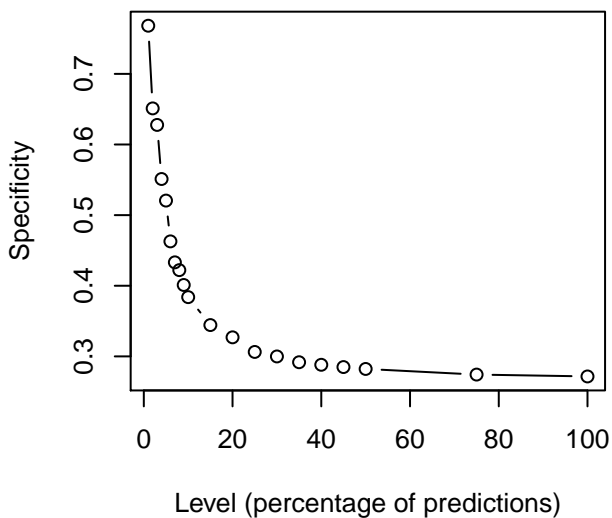

## E2F6

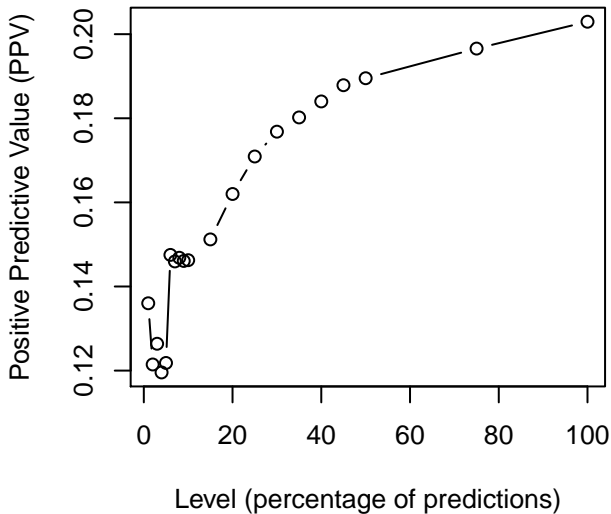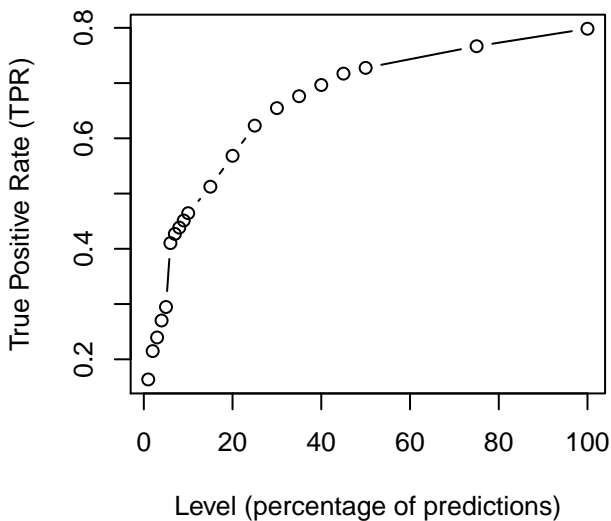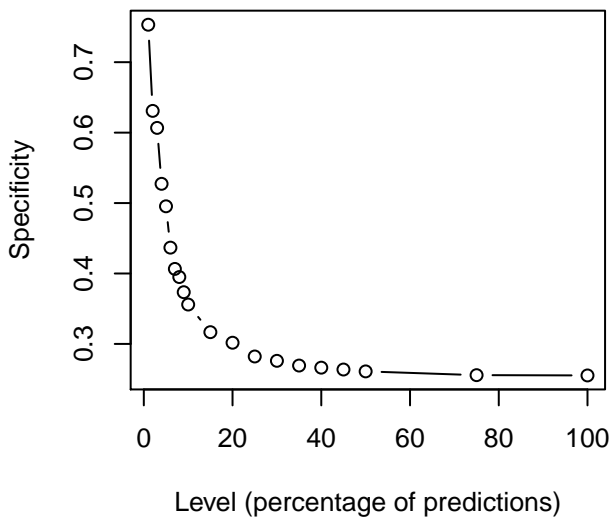

# ELF1

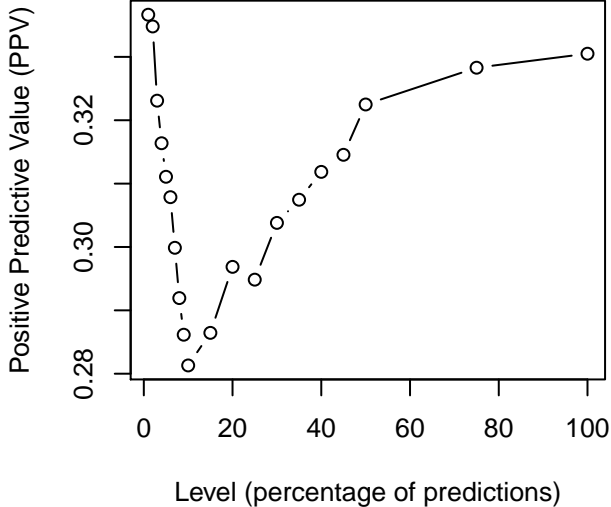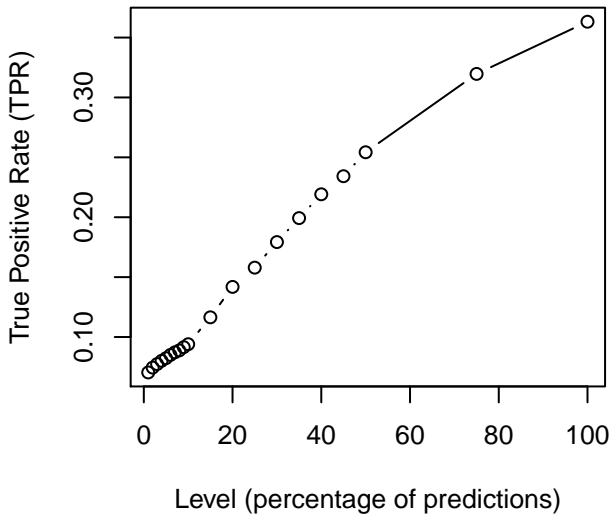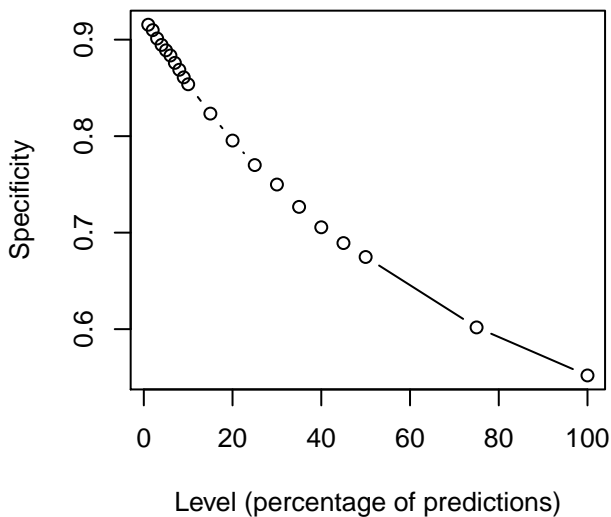

## ETS1

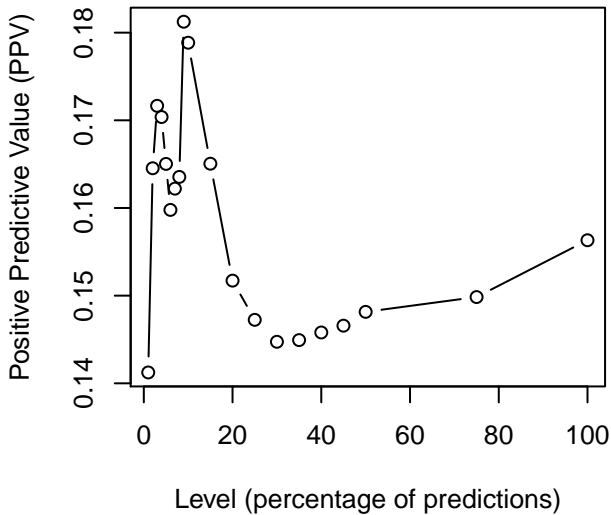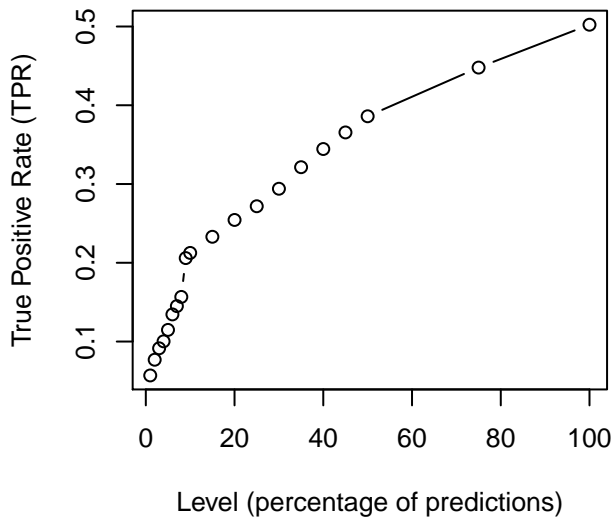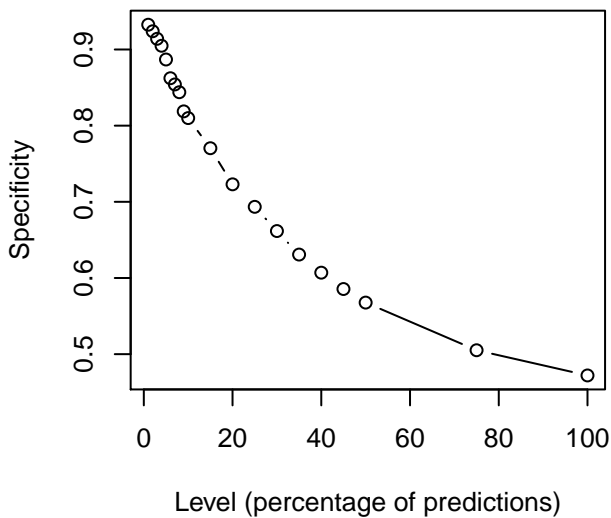

## FOXA1

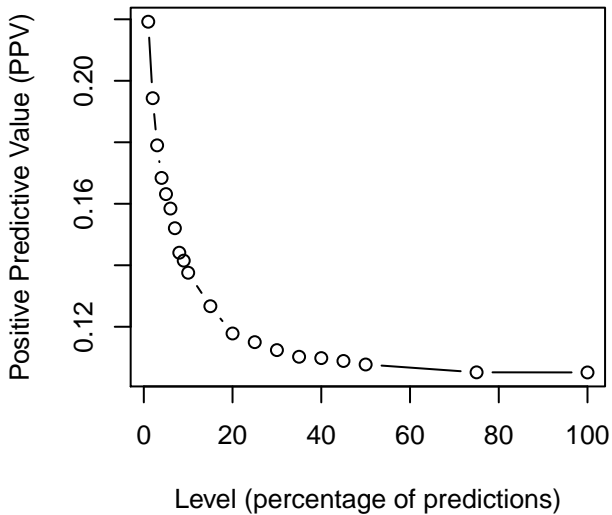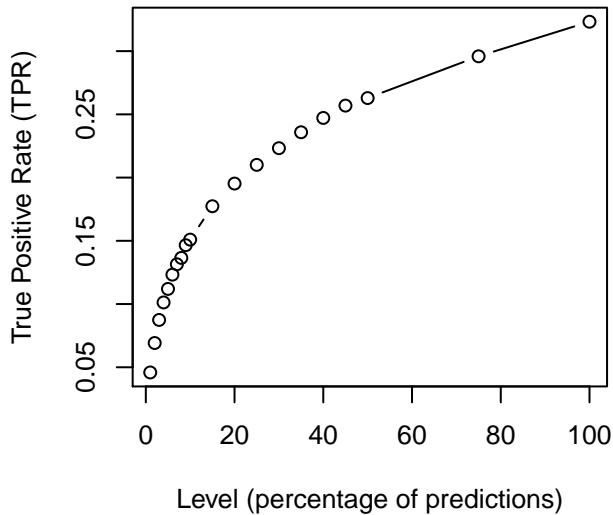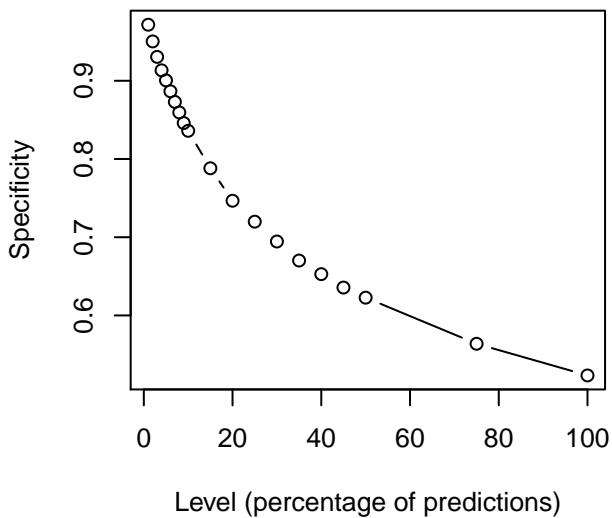

## GATA1

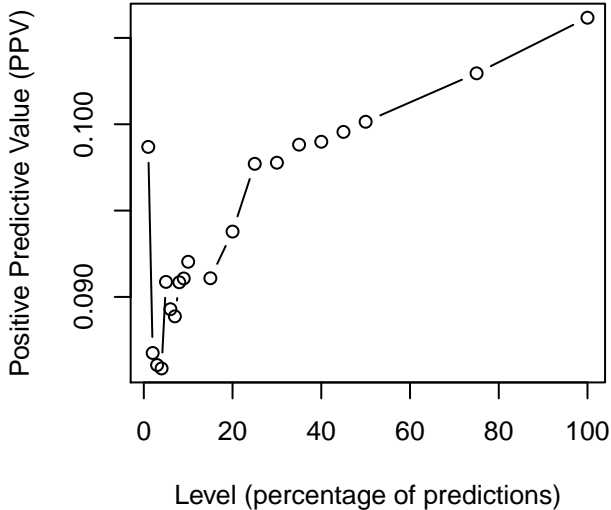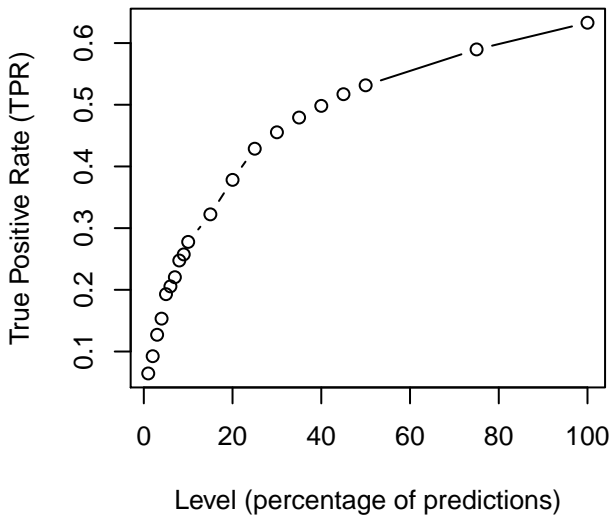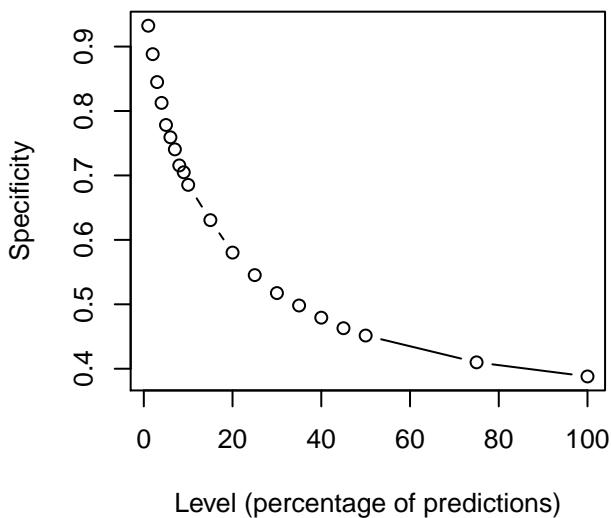

## GATA2

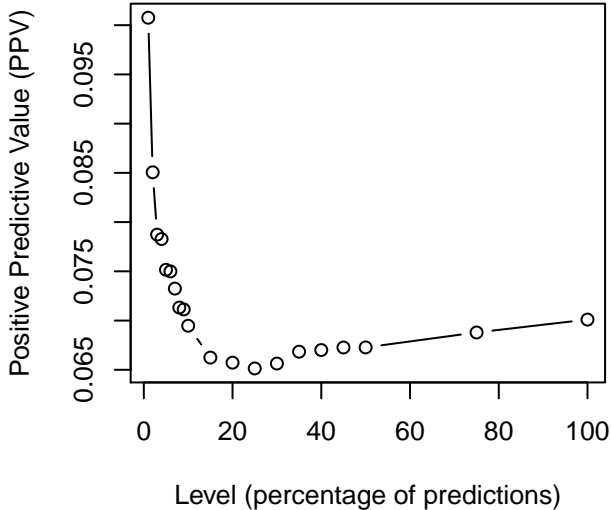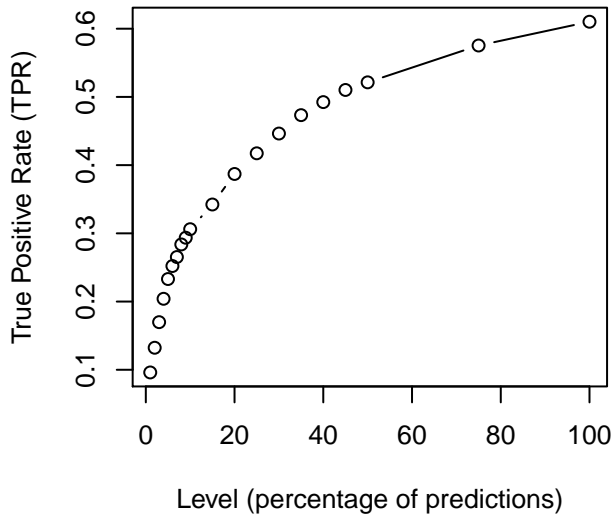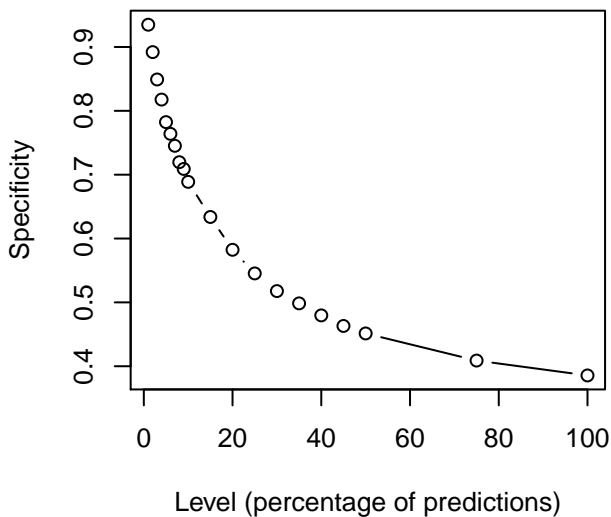

## GATA3

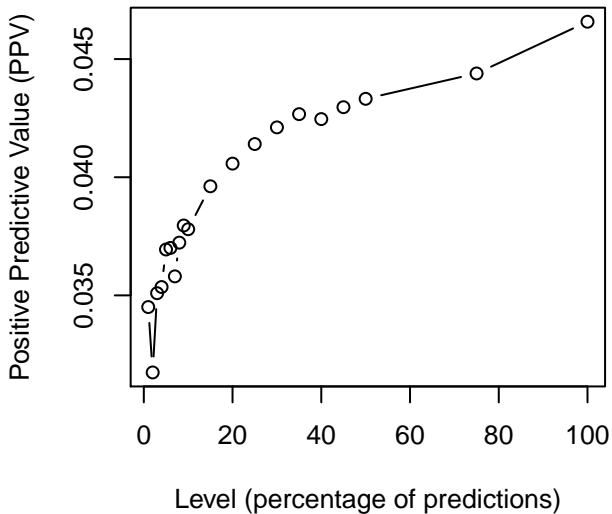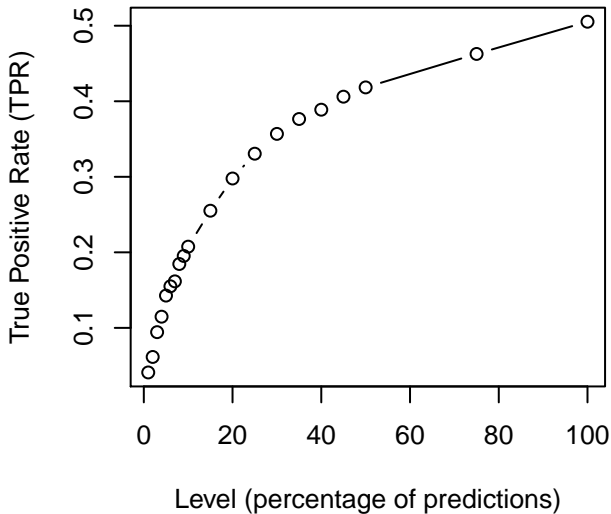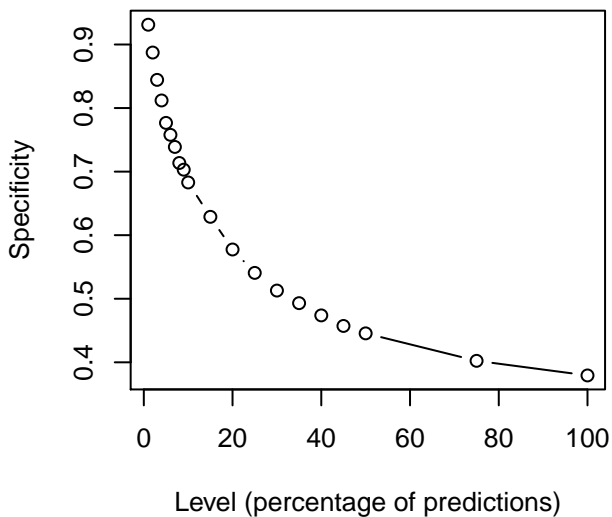

# HEY1

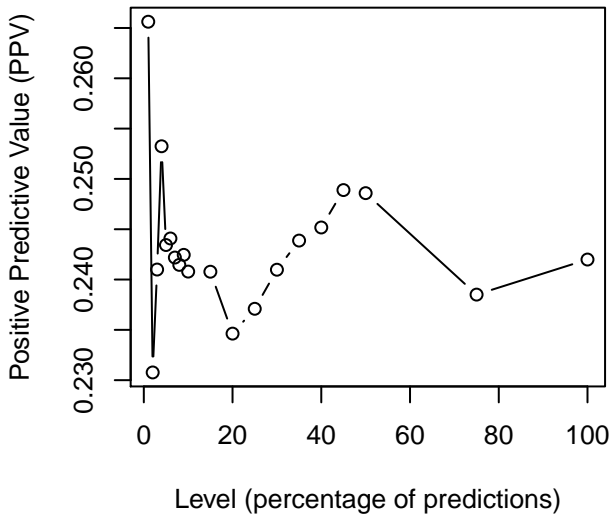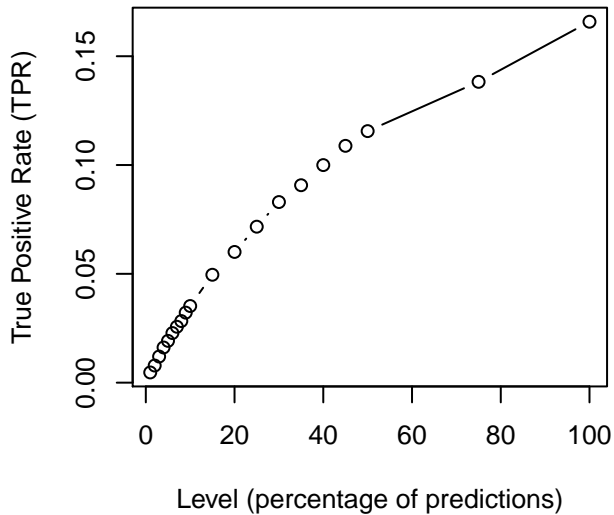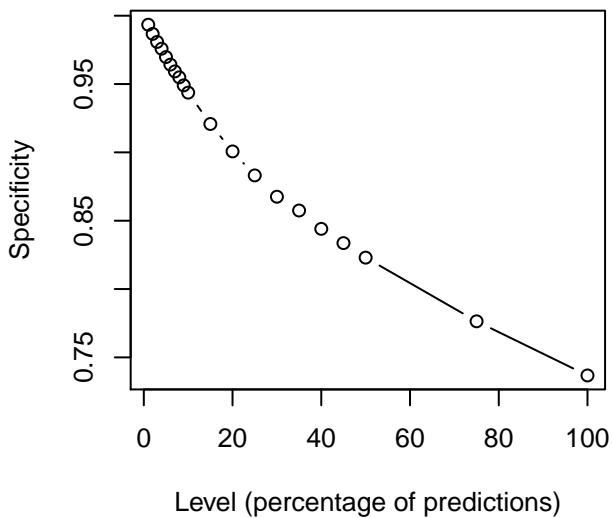

# IRF1

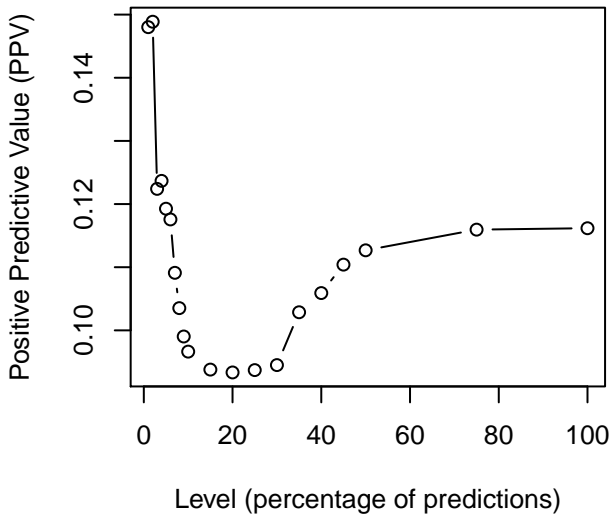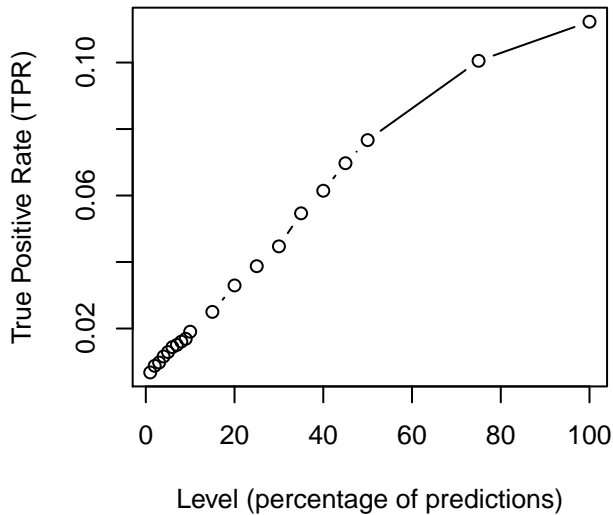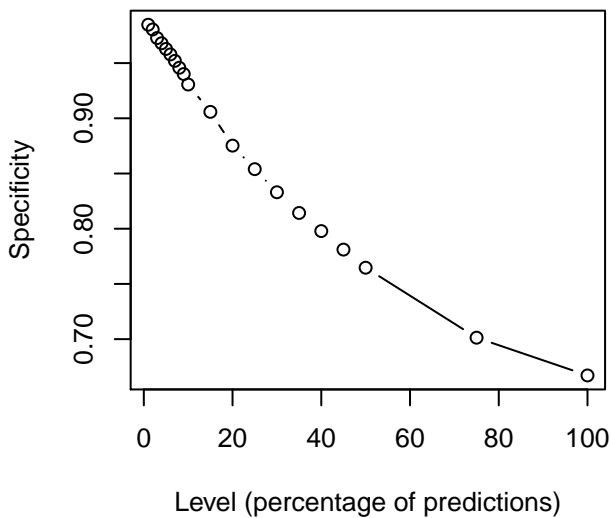

### IRF3

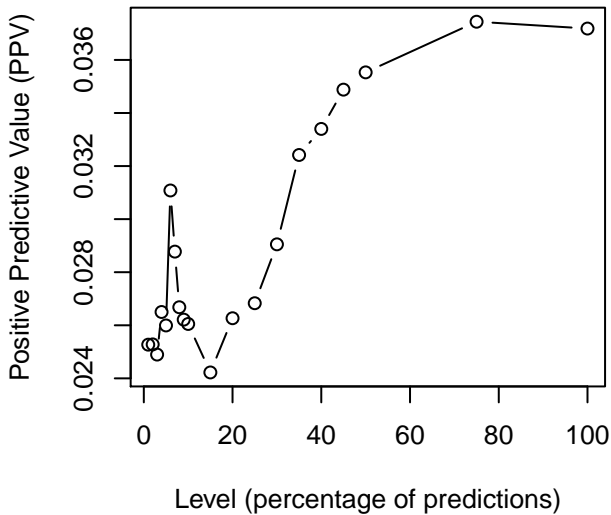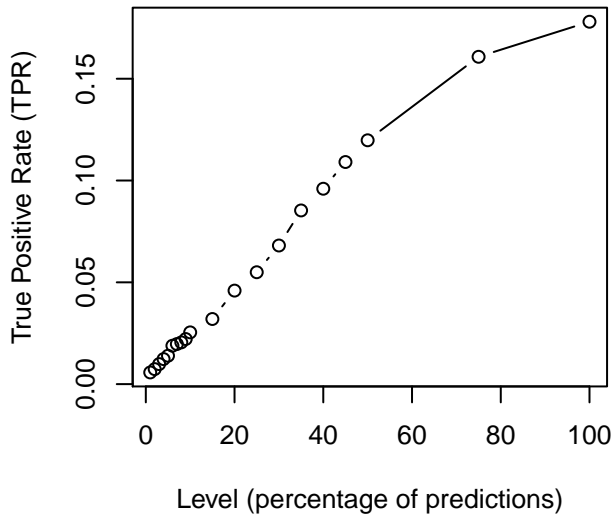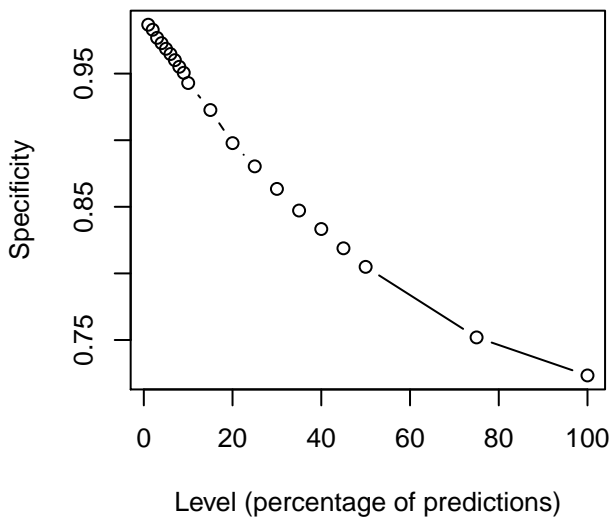

## NANOG

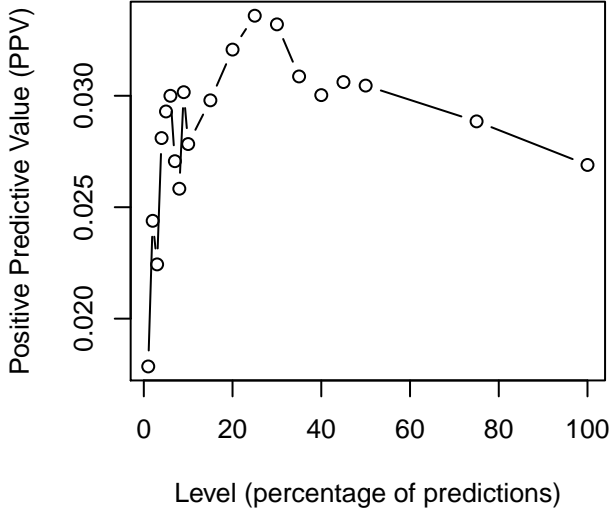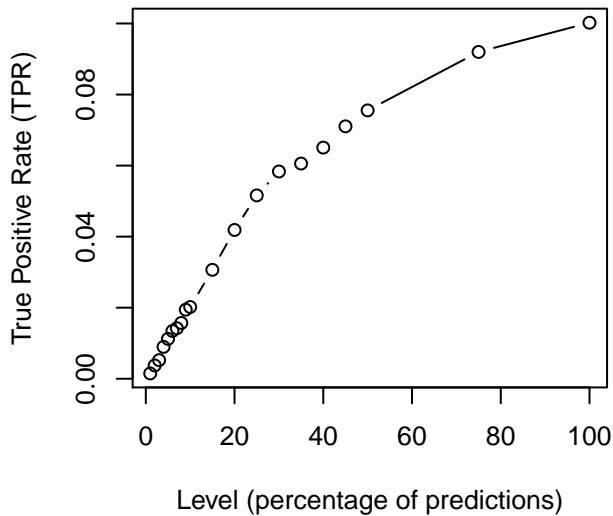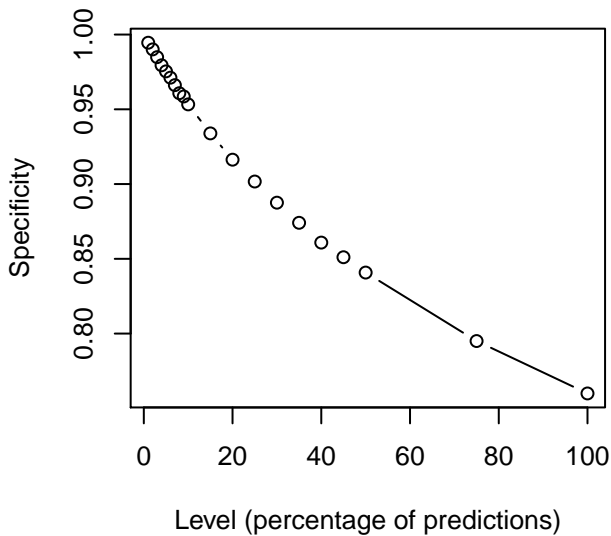

# NFKB

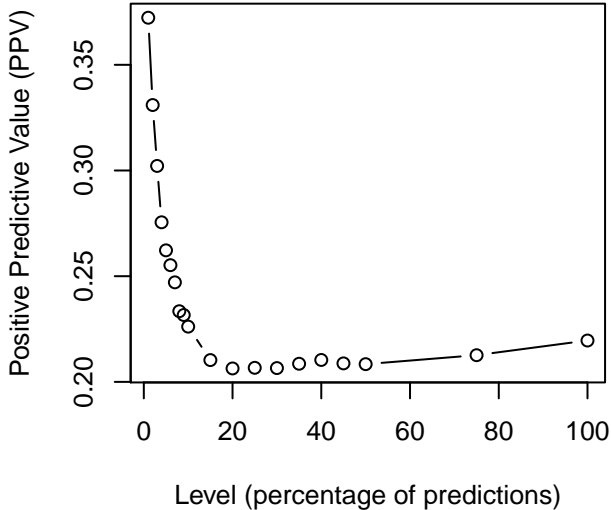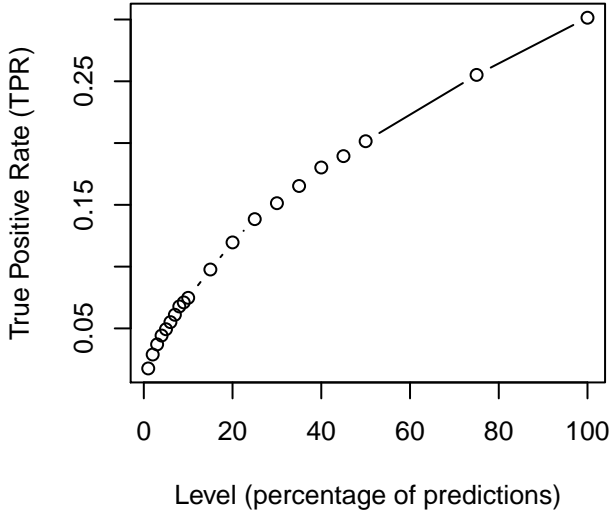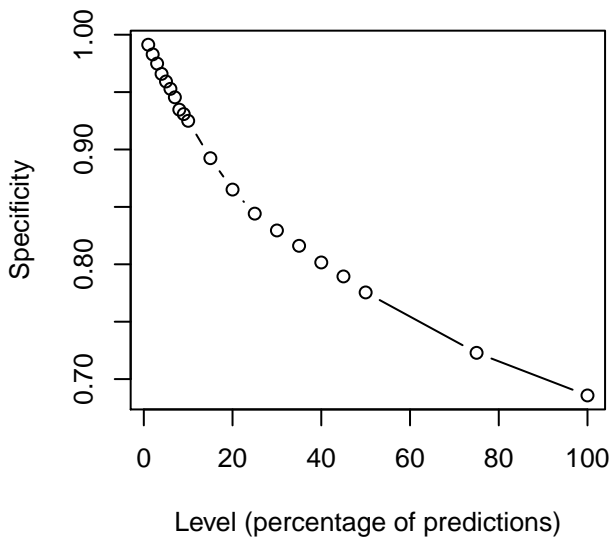

## PAX5

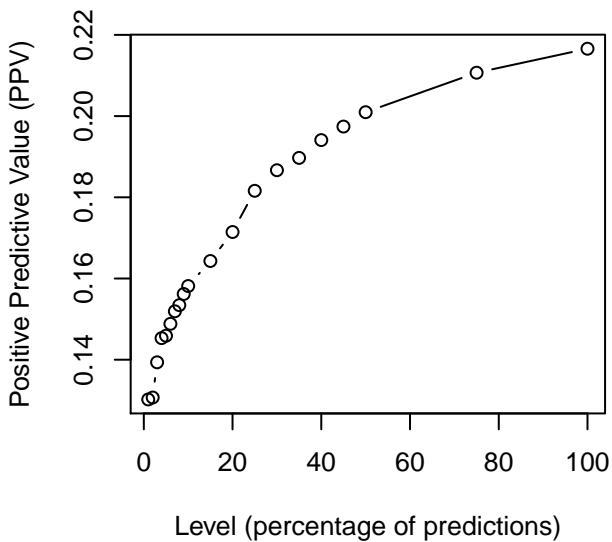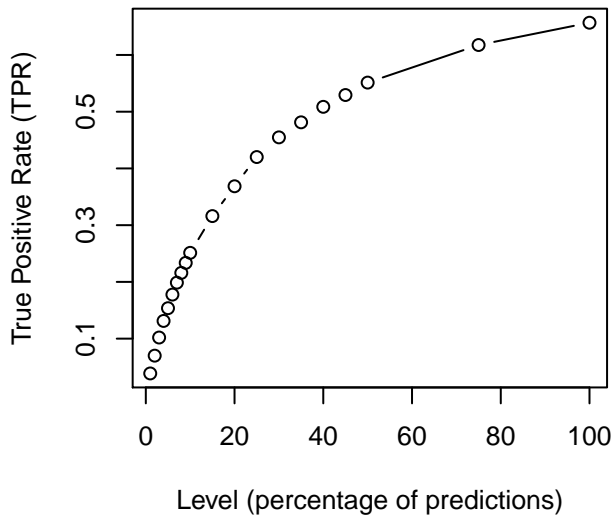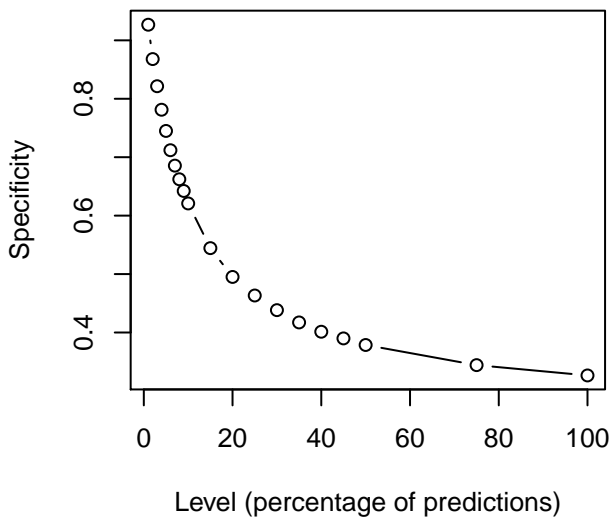

## POU5F1

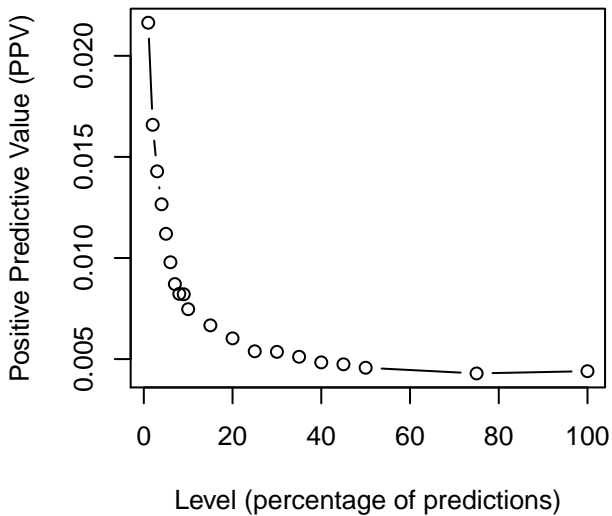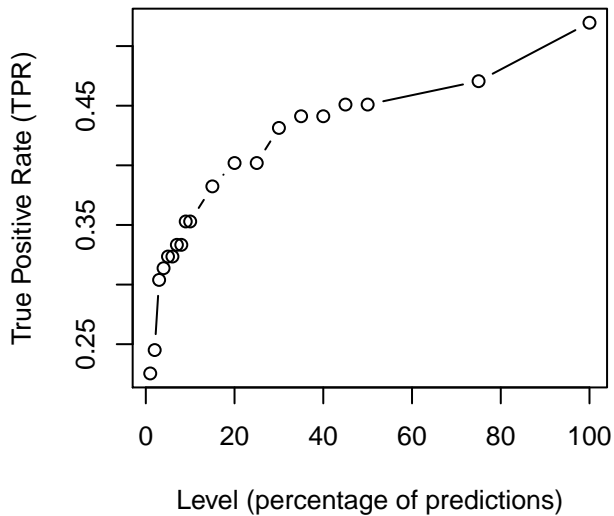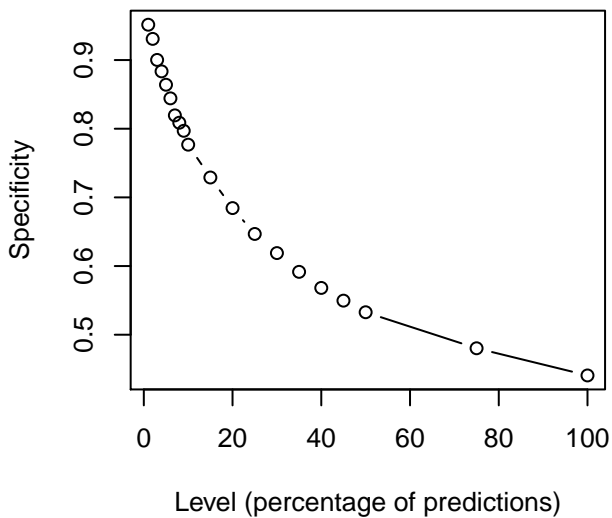

## RXRA

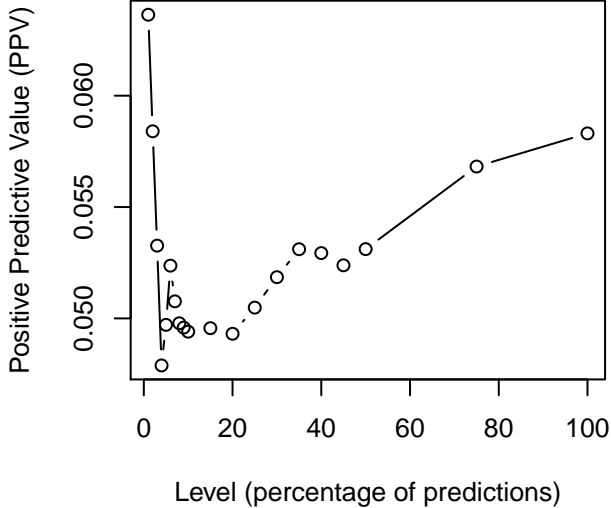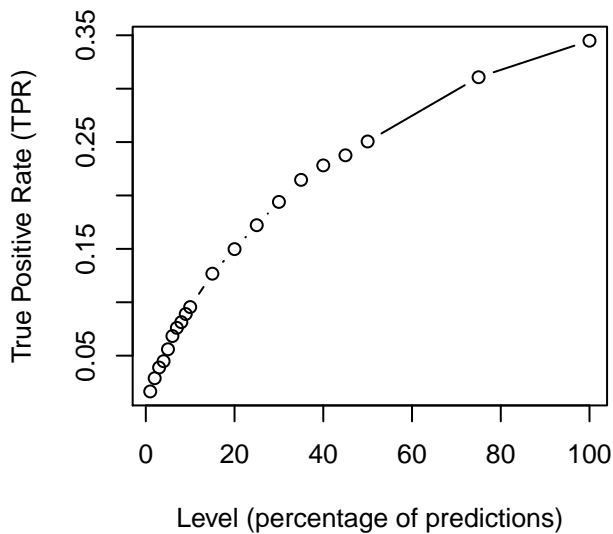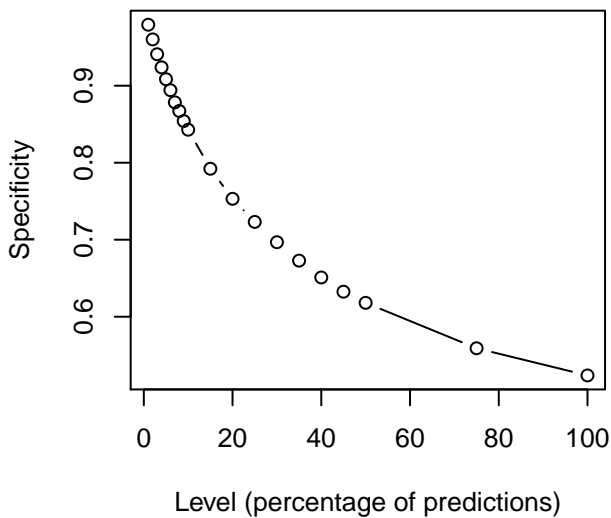

# SP1

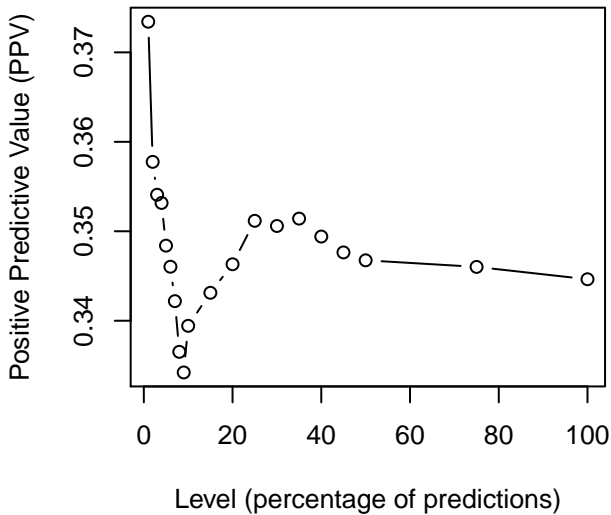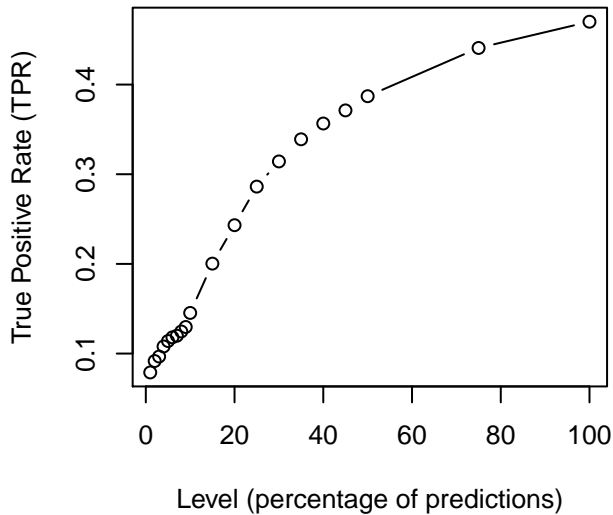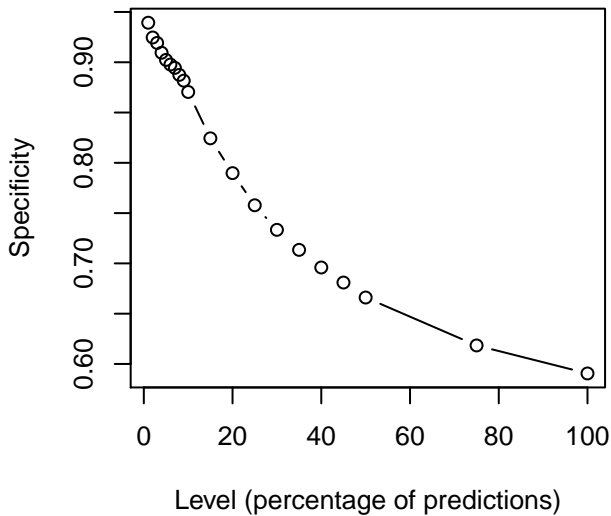

## TFAP2A

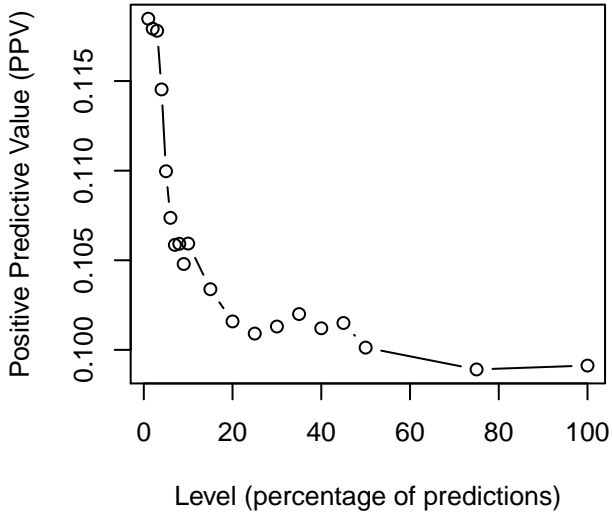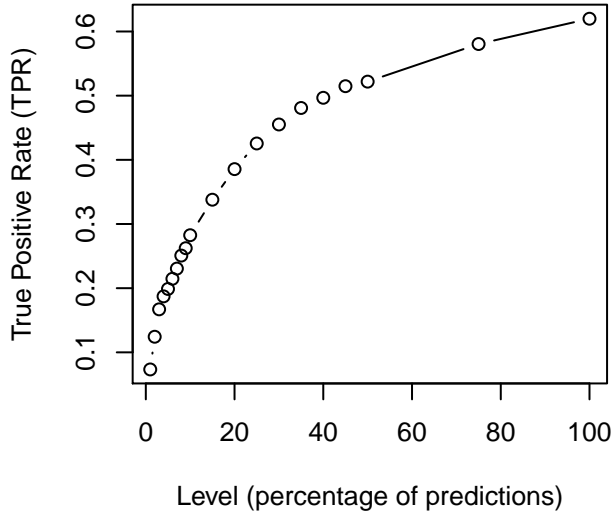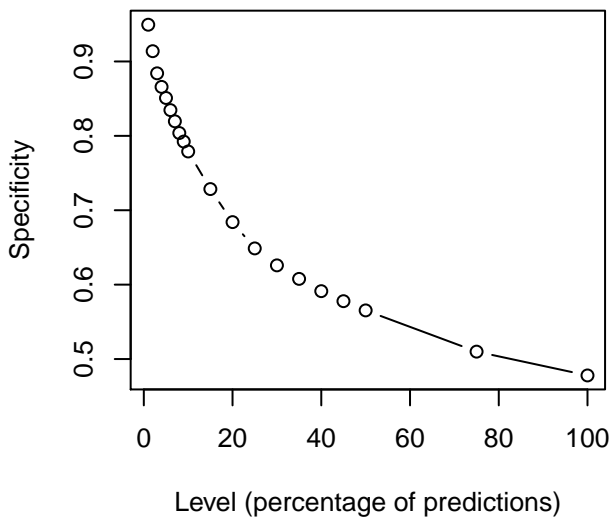

YY1

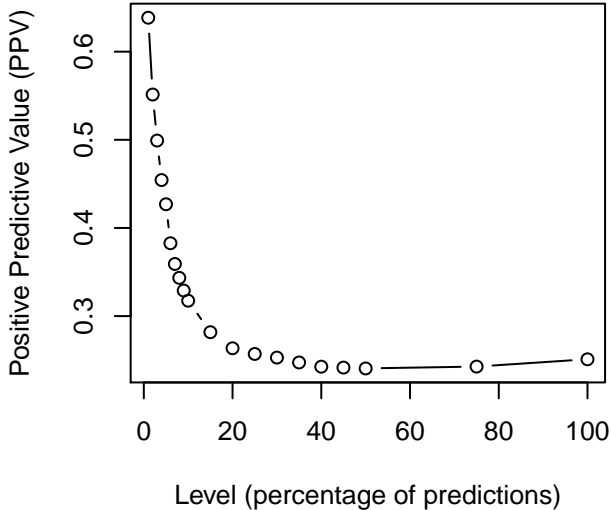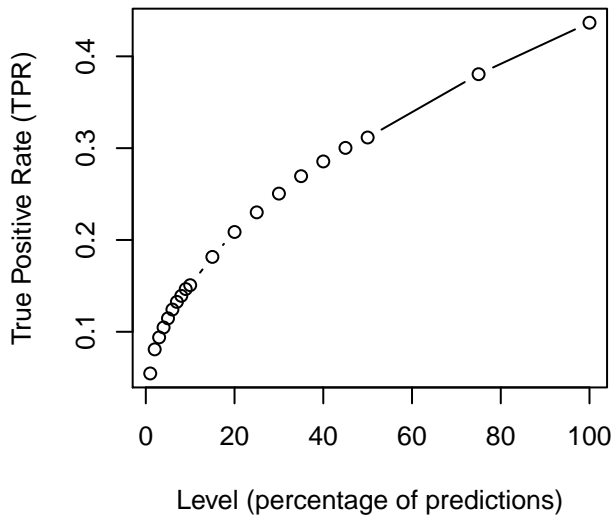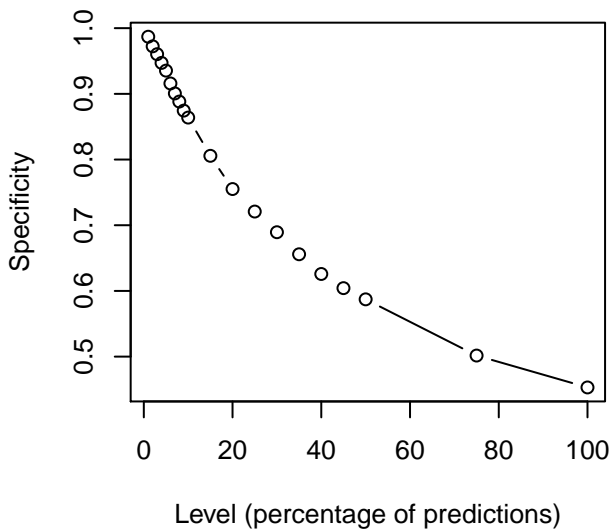

Supplement: Additional file 2 — Prediction validations. In this file, plots of positive predictive value (PPV), true positive rate (TPR) and Specificity are given for all TFs where sufficient experimental data have been made available by the ENCODE project. Validation has been made in each case for all prediction profiles from the 1% top-ranking sites down to 100% of the predictions for conserved binding sites. [file 1752-0509-6-S2-S15-S2.pdf]
