# Supplementary material for: Using potential master regulator sites and paralogous expansion to construct tissue-specific transcriptional networks
Source: BMC Syst Biol. 2012 Dec 12;6(Suppl 2):S15. doi: 10.1186/1752-0509-6-S2-S15 (PMC3521180; doi:10.1186/1752-0509-6-S2-S15)
Supplement: Additional file 3 — Revisited false positives. Venn diagrams of comparing independent experimental datasets for TFBSs within the -1kb regions with each other and the predictions done in this study. [file 1752-0509-6-S2-S15-S3.pdf]

Additional file 2 – Revisited False Positives.

Venn diagrams of comparing independent experimental datasets for TFBSs within the -1kb regions with each other and the predictions done in this study. The blue and the brown circles represent numbers of genes with ChIPseq fragments mapped to the –1kb region obtained from two different cell lines, the purple circle represents the number of predicted TF-target relations according to the presence of at least one potential TFBS in the -1kb region.

Figure 2-1 - c-Jun – target gene relations

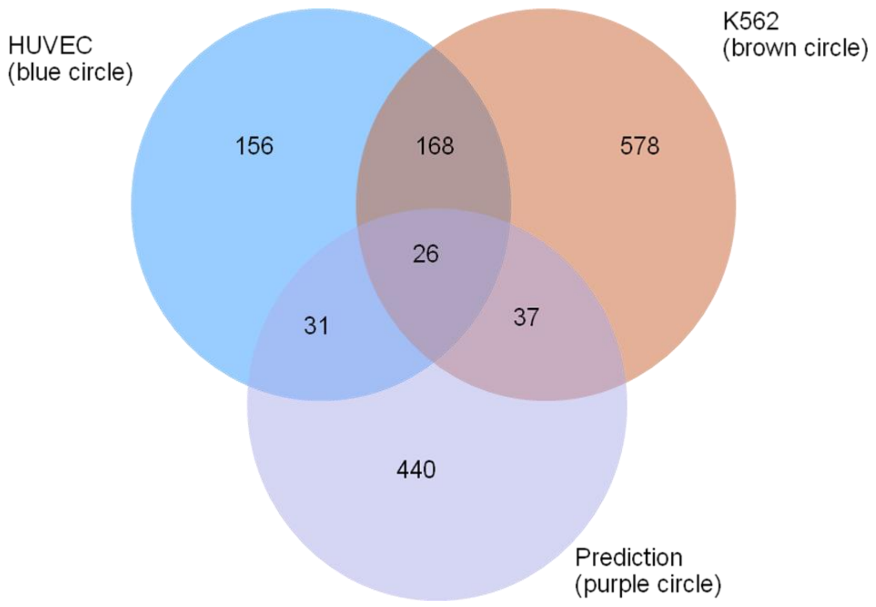

Transcription factor c-Jun – target gene relations using ChIPseq data from HUVEC (human umbilical vein endothelial cells) or K562 cells (a myeloid blood cell line), in comparison with relations obtained from predicted 1% top-ranked high-affinity, conserved sites.

ENCODE data sets: (a)wgEncodeSydhTfbsHuvecCjunStdPk.narrowPeak; (b) wgEncodeSydhTfbsK562CjunStdPk.narrowPeak

Figure 2-2 - c-Fos – target gene relations

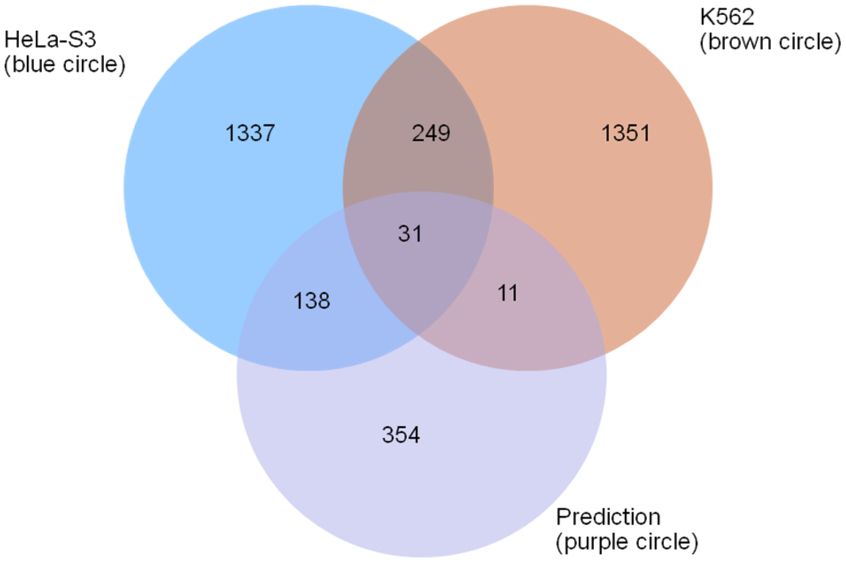

Transcription factor c-Fos – target gene relations using ChIPseq data from HeLa-S3 (an endothelial, cervical carcinoma cell line) or K562 cells (a myeloid blood cell line), in comparison with relations obtained from predicted 1% top-ranked high-affinity, conserved sites.

ENCODE data sets: (a) wgEncodeSydhTfbsHelas3CfosStdPk.narrowPeak; (b) wgEncodeSydhTfbsK562CfosStdPk.narrowPeak

Figure 2-3 - c-Myc – target gene relations

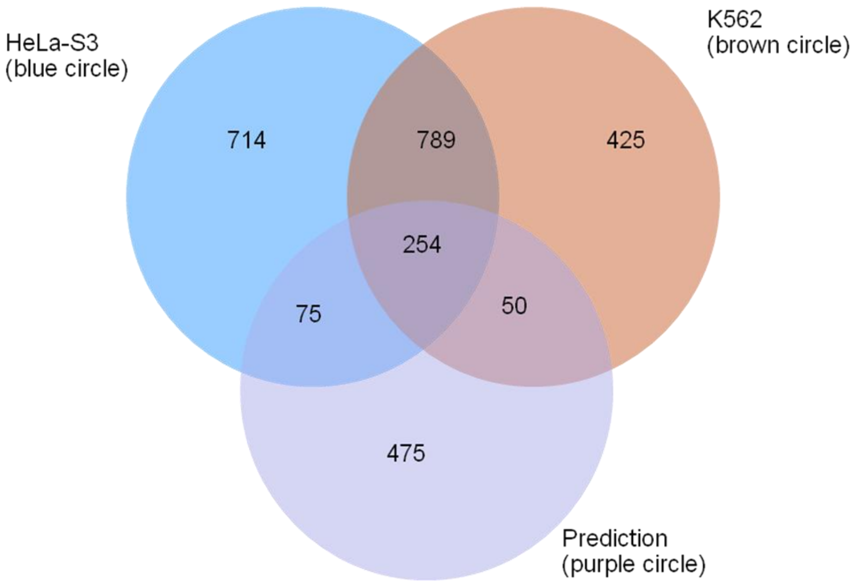

Transcription factor c-Myc – target gene relations using ChIPseq data from HeLa-S3 (an endothelial, cervical carcinoma cell line) or K562 cells (a myeloid blood cell line), in comparison with relations obtained from predicted 1% top-ranked high-affinity, conserved sites.

ENCODE data sets: (a) wgEncodeSydhTfbsHelas3CmycStdPk.narrowPeak; (b) wgEncodeSydhTfbsK562CmycStdPk.narrowPeak

Figure 2-4 - Max – target gene relations

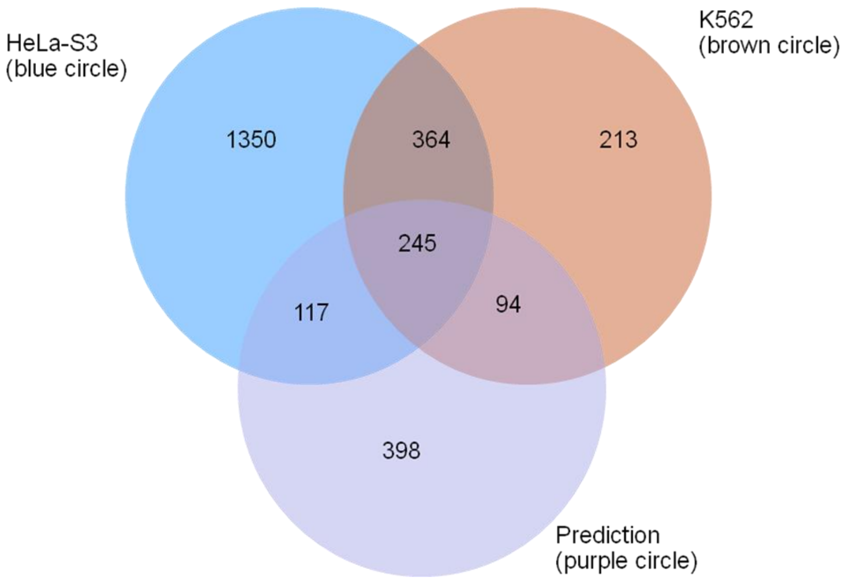

Transcription factor Max – target gene relations using ChIPseq data from HeLa-S3 (an endothelial, cervical carcinoma cell line) or K562 cells (a myeloid blood cell line), in comparison with relations obtained from predicted 1% top-ranked high-affinity, conserved sites.

ENCODE data sets: (a) wgEncodeSydhTfbsHelas3MaxStdPk.narrowPeak; (b) wgEncodeSydhTfbsK562MaxStdPk.narrowPeak
