# Supplementary material for: Using potential master regulator sites and paralogous expansion to construct tissue-specific transcriptional networks
Source: BMC Syst Biol. 2012 Dec 12;6(Suppl 2):S15. doi: 10.1186/1752-0509-6-S2-S15 (PMC3521180; doi:10.1186/1752-0509-6-S2-S15)
Supplement: Additional file 4 — Degree statistics. The table indicates the average out-degree and in-degree of TF genes as well as the average in-degree of nonTF genes (NTF) for both the reconstructed transcriptional networks (reference network and tissue-specific networks) as well as the transcriptional network expanded by related TFs. It also shows the ratios of the corrresponding values for the expanded and the non-expanded networks (eTN/TN). [file 1752-0509-6-S2-S15-S4.pdf]

Network	Transcriptional networks (TN)				Expanded transcriptional networks (eTN)				eTN/TN		
	TF_outdeg	TF_indeg	NTF_indeg	TF_in/ NTF_in	TF_outdeg	TF_indeg	NTF_indeg	TF_in/ NTF_in	TF_outdeg	TF_indeg	NTF_indeg
Reference	628.2	26.5	17.5	1.51	982	62	45.2	1.37	1.56	2.34	2.58
Brain	488.3	22.1	13.6	1.63	797.6	51.7	35	1.48	1.63	2.34	2.57
Heart	300.8	13.2	8.8	1.50	508.9	30.6	21	1.46	1.69	2.32	2.39
Kidney	400.5	18.3	11.4	1.61	653.9	41.8	27.2	1.54	1.63	2.28	2.39
Liver	350.1	15.2	9.8	1.55	562.3	33.8	22.8	1.48	1.61	2.22	2.33
Ovary	326.1	15	9.6	1.56	507.2	33.5	21.7	1.54	1.56	2.23	2.26
Prostate	376.5	17	10.5	1.62	591.9	38.4	24.9	1.54	1.57	2.26	2.37
Spleen	210.9	9.2	6.2	1.48	338.6	20.2	12.8	1.58	1.61	2.20	2.06
Testis	422.8	17.6	10.9	1.61	685.5	42.1	28.3	1.49	1.62	2.39	2.60
Average	*389.36*	*17.12*	*10.92*	*1.56*	*625.32*	*39.34*	*26.54*	*1.50*	*1.61*	*2.29*	*2.39*

TF_outdeg: out-degree of TF genes
TF_indeg: in-degree of TF genes
NTF_indeg: in-degree of nonTF genes

TF_in/ NTF_in
0.91
0.91
0.97
0.96
0.96
0.99
0.95
1.06
0.92
0.96
