# Supplementary material for: Using potential master regulator sites and paralogous expansion to construct tissue-specific transcriptional networks
Source: BMC Syst Biol. 2012 Dec 12;6(Suppl 2):S15. doi: 10.1186/1752-0509-6-S2-S15 (PMC3521180; doi:10.1186/1752-0509-6-S2-S15)
Supplement: Additional file 5 — Degree distributions of tissue-specific transcriptional networks. Inverse cumulative in- and out-degree distributions of the tissue-specific transcriptional networks (TTNs) before and after paralogous expansions. [file 1752-0509-6-S2-S15-S5.pdf]

Additional File 5

Figure 5-1 - Inverse cumulative in-degree distribution of the transcriptional networks before expansion

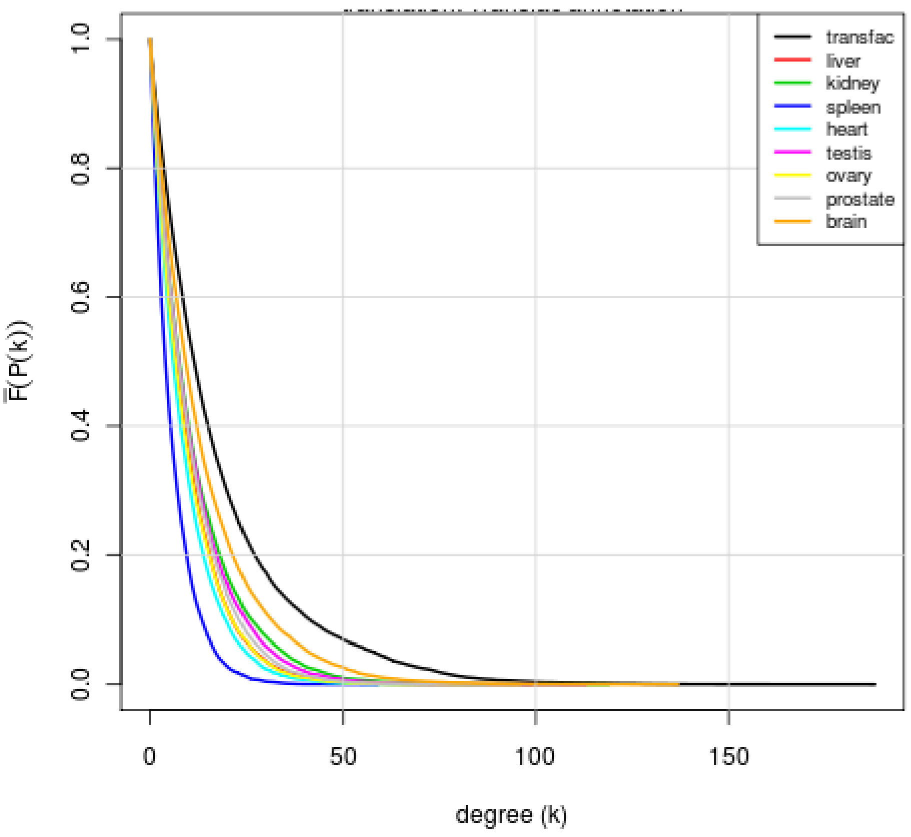

Inverse cumulative in-degree distribution of the transcriptional networks. The reference network is colored black (labeled “transfac”). The color code for the tissue-specific instances is given in the legend.

Figure 5-2 - Inverse cumulative out-degree distribution of the transcriptional networks before expansion

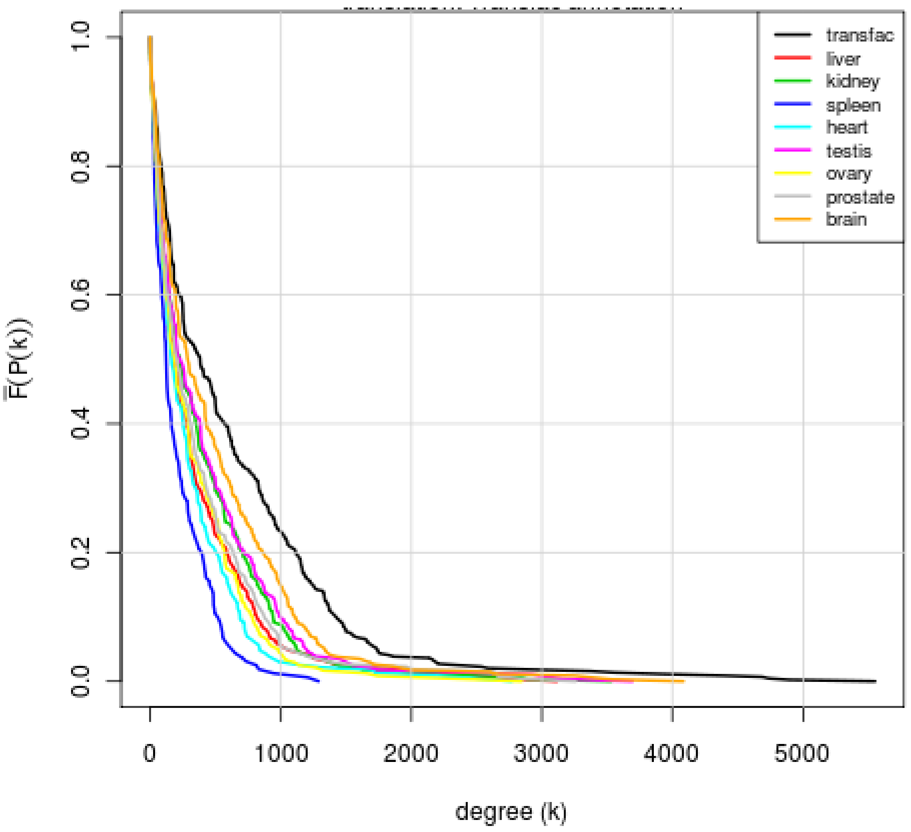

Inverse cumulative out-degree distribution of the transcriptional networks. The reference network is colored black (labeled "transfac"). The color code for the tissue-specific instances is given in the legend.

Figure 5-3 - Inverse cumulative in-degree distribution after paralogous expansion

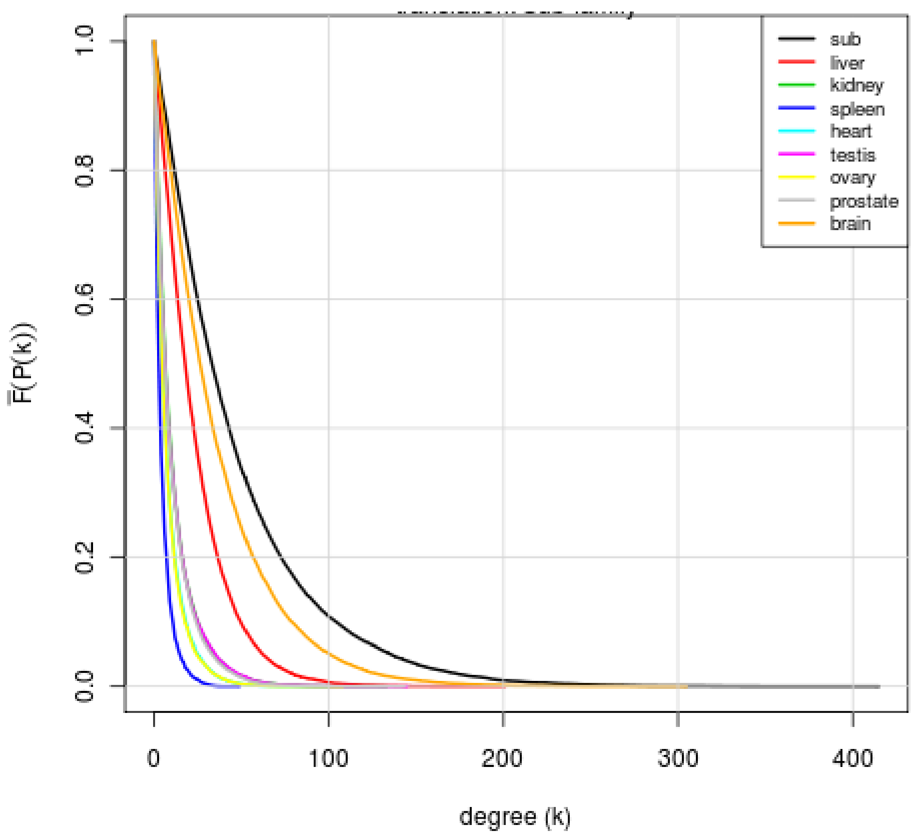

Inverse cumulative in-degree distribution of the expanded transcriptional networks. The reference network is colored black (labeled "sub"). The color code for the tissue-specific instances is given in the legend.

Figure 5-4 – Inverse cumulative out-degree distribution after paralogous expansion

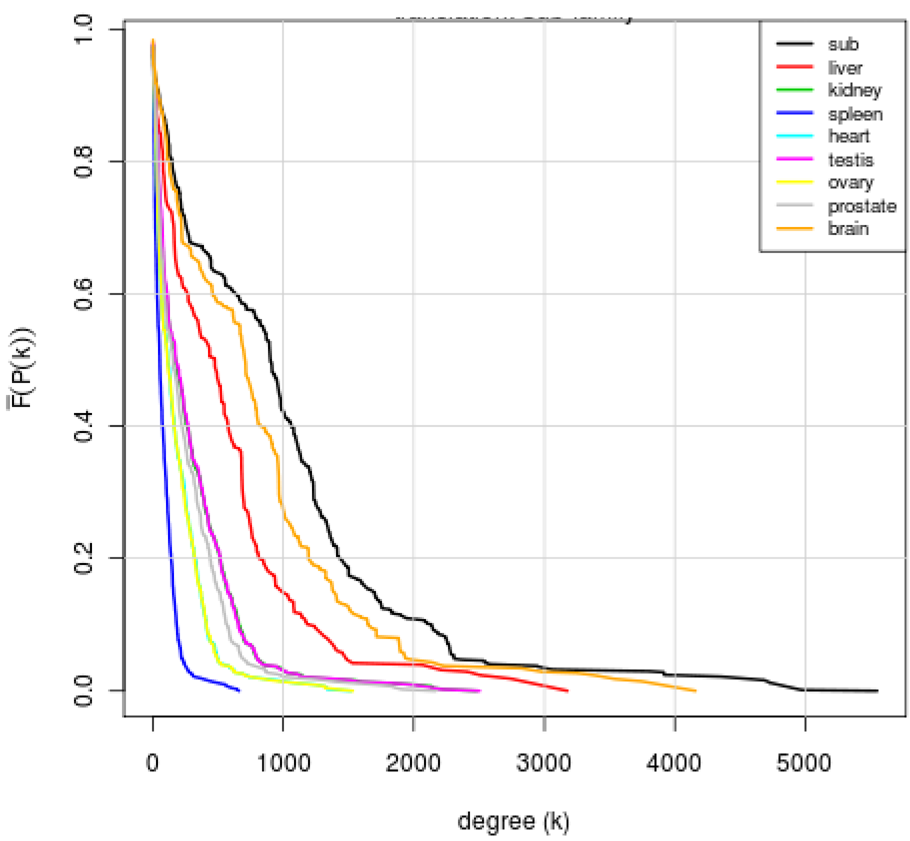

Inverse cumulative out-degree distribution of the expanded transcriptional networks. The reference network is colored black (labeled "sub"). The color code for the tissue-specific instances is given in the legend.
